# Multi-organ proteomics identifies oxidative stress pathways underlying genetic and pharmacological SGLT2 suppression

**DOI:** 10.1016/j.redox.2026.104472

**Published:** 2026-11-03

**Authors:** Marta Parente Nascimento, Ilyas Majid, Anu Arthy Murugan Karunagaran, Sophia Ginns, Dorothee Heger, Cian Sutcliffe, Ayesha Roked, John McDonald, Thong Huy Cao, Paulene Quinn, Colleen B. Maxwell, Luke A. Baker, Melanie J. Davies, Donald JL. Jones, Leong L. Ng, Elina Akalestou

**Affiliations:** a Division of Cardiovascular Sciences, School of Medical Sciences, College of Life Sciences, University of Leicester, Leicester, UK; b Division of Respiratory Sciences, College of Life Sciences, University of Leicester, Leicester, UK; c National Institute for Health and Care Research Biomedical Research Centre Leicester, UK; d Leicester Van Geest Multi-OMICS Facility, University of Leicester, Leicester, UK; e British Heart Foundation Centre of Research Excellence, College of Life Sciences, University of Leicester, Leicester, UK; f Diabetes Research Centre, College of Life Sciences, University of Leicester, Leicester General Hospital, Leicester, UK; g Division of Cancer Sciences, College of Life Sciences, University of Leicester, Leicester, UK; h Section of Cell Biology and Functional Genomics, Division of Diabetes, Endocrinology and Metabolism, Department of Metabolism, Digestion and Reproduction, Imperial College London, London, UK

**Keywords:** SGLT2, Proteomics, System, Oxidative stress

## Abstract

**Background:**

Sodium–glucose cotransporter 2 (SGLT2) inhibitors provide clinical benefits beyond glycaemic control, although the underlying systemic mechanisms remain incompletely understood. *Methods*: We performed proteomic profiling across eight metabolically relevant tissues and plasma in high-fat diet-fed mice with either genetic deletion (SGLT2^−/−^) or pharmacological inhibition (dapagliflozin) of SGLT2. Differentially expressed proteins were analysed using Gene Ontology, KEGG pathway enrichment, and integrative clustering. Cross-species comparison was performed using plasma and skeletal-muscle proteomic datasets from participants with type 2 diabetes treated with dapagliflozin.

**Results:**

Both genetic and pharmacological SGLT2 inhibition induced widespread tissue-specific proteomic remodelling, particularly in liver, adrenal glands, and adipose tissue. Enrichment analyses consistently identified oxidative stress, mitochondrial function, and metabolic pathways as dominant processes. Shared protein signatures across conditions were strongly linked to reactive oxygen species regulation, mitochondrial respiration, and glutathione metabolism. Tissue-specific analyses revealed divergent responses, including increased oxidative pathways in liver and adrenal tissue, but reduced oxidative signalling in adipose tissue and heart. Cross-species analyses identified overlapping oxidative stress-, mitochondrial- and energy-metabolism pathways in human plasma and skeletal muscle. *Conclusions*: SGLT2 inhibition induces coordinated multi-organ proteomic remodelling characterised by conserved oxidative stress and mitochondrial signatures, supporting a systemic mechanism underlying the pleiotropic benefits of SGLT2 inhibitors.

## Highlights

• Multi-organ proteomics identifies oxidative stress regulation as a central response to SGLT2 inhibition.
• Genetic and pharmacological SGLT2 inhibition share a conserved oxidative stress proteomic signature across tissues and plasma.
• Liver and adrenal glands exhibit the most extensive redox and metabolic remodelling following SGLT2 inhibition.
• Mitochondrial respiration, glutathione metabolism, and ROS-associated pathways are consistently altered across organs.
• Conserved oxidative stress signatures in human plasma and skeletal muscle support clinical translation of the findings.

## Graphical abstract

**Figure:**
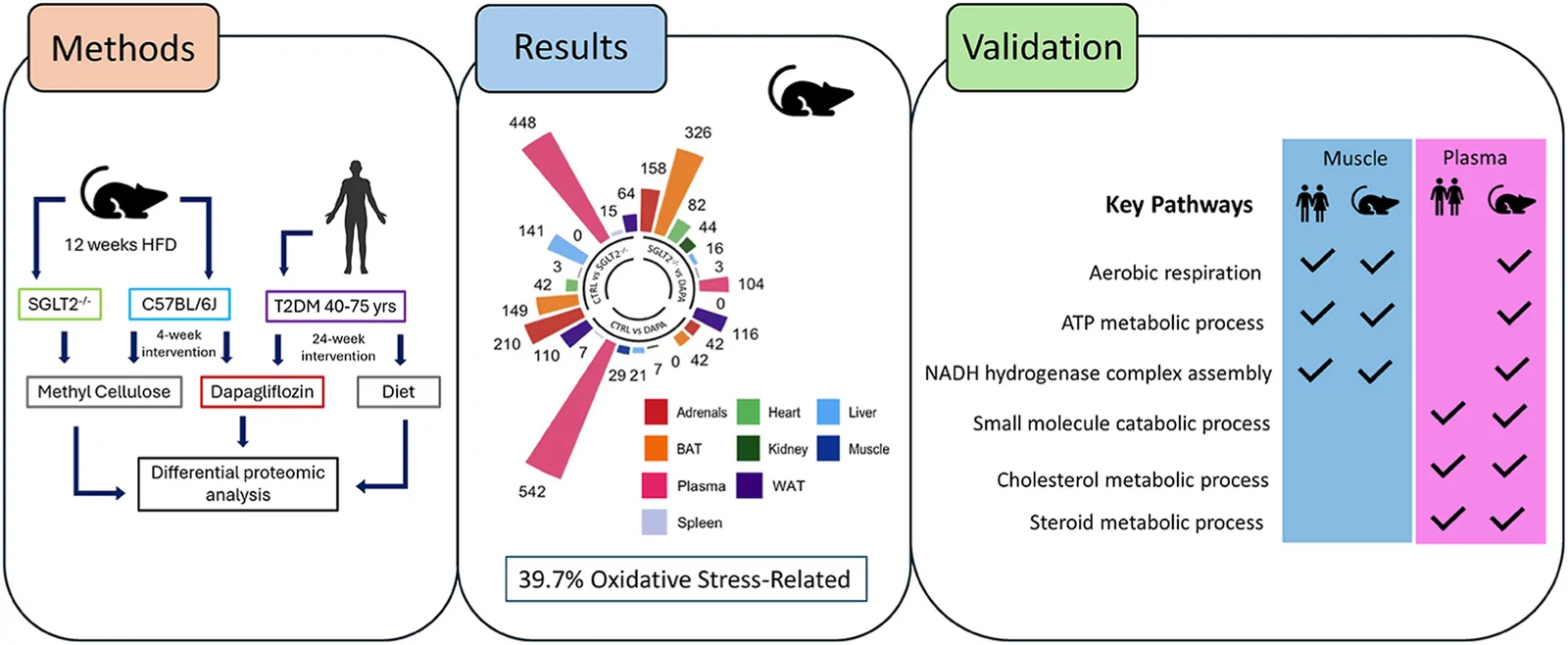

## 1 Introduction

Metabolic diseases, including Type 2 Diabetes (T2D), cardiovascular disease, chronic kidney disease, and metabolic dysfunction-associated steatotic liver disease, represent a major and growing global health burden. These conditions frequently coexist and are characterised by progressive dysfunction across multiple organs, including the liver, kidney, heart, skeletal muscle, and adipose tissue. Despite differences in clinical presentation, these diseases share common underlying mechanisms, including metabolic dysregulation, chronic low-grade inflammation, oxidative stress, and impaired cellular homeostasis, which together contribute to progressive tissue dysfunction and systemic disease progression [1], [2]. Increasing evidence suggests that these pathological processes are interconnected across organs, resulting in coordinated molecular and metabolic alterations that drive multimorbidity.

Given the shared pathophysiology underlying metabolic disease, therapies that confer protection across multiple organs have emerged as important therapeutic strategies. One such class is the sodium–glucose cotransporter 2 (SGLT2) inhibitors, initially developed for the treatment of T2D. SGLT2 is predominantly expressed in the renal proximal tubule, where it mediates the majority of glucose reabsorption. Pharmacological inhibition of SGLT2 reduces renal glucose reabsorption and improves glycaemic control [3]. However, beyond their glucose-lowering effects, SGLT2 inhibitors have demonstrated substantial cardiovascular and renal benefits, alongside emerging therapeutic effects in liver and metabolic disease [4]. Notably, these benefits are observed across diverse patient populations and disease states, suggesting that SGLT2 inhibition modulates fundamental biological pathways extending beyond glycaemic regulation alone [4], [5]. Importantly, accumulating experimental evidence indicates that some cardiovascular actions of SGLT2 inhibitors can occur independently of SGLT2 itself. Studies using genetic SGLT2-deficient models have demonstrated pharmacological effects that are not reproduced by SGLT2 deletion alone, including cardioprotection associated with modulation of inflammatory, oxidative stress and NHE1–NO signalling pathways [6], [7], [8], [9], [10]. These observations suggest that the beneficial actions of SGLT2 inhibitors may comprise both SGLT2-dependent and SGLT2-independent mechanisms and provide a rationale for comparing genetic SGLT2 deletion with pharmacological SGLT2 inhibition.

Growing interest in the pleiotropic effects of SGLT2 inhibition has prompted investigation of SGLT2 biology beyond glycaemic control. Alongside clinical and experimental use of pharmacological inhibitors, murine models of genetic SGLT2 deficiency have been widely employed to define the function of the transporter [11], [12]. However, owing to the practical and ethical limitations associated with obtaining multiple tissue biopsies in humans, it remains unclear whether the molecular effects of SGLT2 inhibition are tissue-specific or reflect coordinated systemic responses across metabolically relevant organs.

Here, we performed comprehensive proteomic profiling across eight metabolically relevant tissues and plasma in high-fat diet-fed mice with either genetic deletion or pharmacological inhibition of SGLT2. Three complementary comparisons were analysed, each addressing a distinct biological question. Comparison of SGLT2-null mice with vehicle-treated controls defines the molecular consequences associated with genetic loss of SGLT2, whereas comparison of dapagliflozin-treated mice with controls captures the combined consequences of pharmacological SGLT2 inhibition and potential SGLT2-independent drug actions. Finally, the comparison of SGLT2-null and dapagliflozin-treated mice identifies molecular responses that distinguish genetic SGLT2 loss from pharmacological inhibition, thereby highlighting candidate SGLT2-independent effects of dapagliflozin while acknowledging potential differences arising from lifelong genetic deletion versus short-term pharmacological treatment.

## 2 Results

### 2.1 Liver and adrenals exhibit the most pronounced proteomic changes in SGLT2^−/−^ vs. control mice

HFD-fed C56Bl/6J mice treated with either dapagliflozin or vehicle, and SGLT2 null mice were used (Fig. 1a). Eight organs and blood plasma were selected and harvested for differential proteomic analysis, including the heart, liver, adrenals, kidney, muscle, white adipose tissue (WAT), spleen and brown adipose tissue (BAT) (Fig. 1b and c). All tissues were prepared for the liquid chromatography-tandem mass spectrometry (LC-MS/MS), beginning with lysis of frozen tissue and enzymolysis to release free peptides, followed by freeze drying and analysed by the Waters G2S LC-MS/MS for subsequential differential proteomic analysis (Fig. 1d and e).

**Fig. 1:**
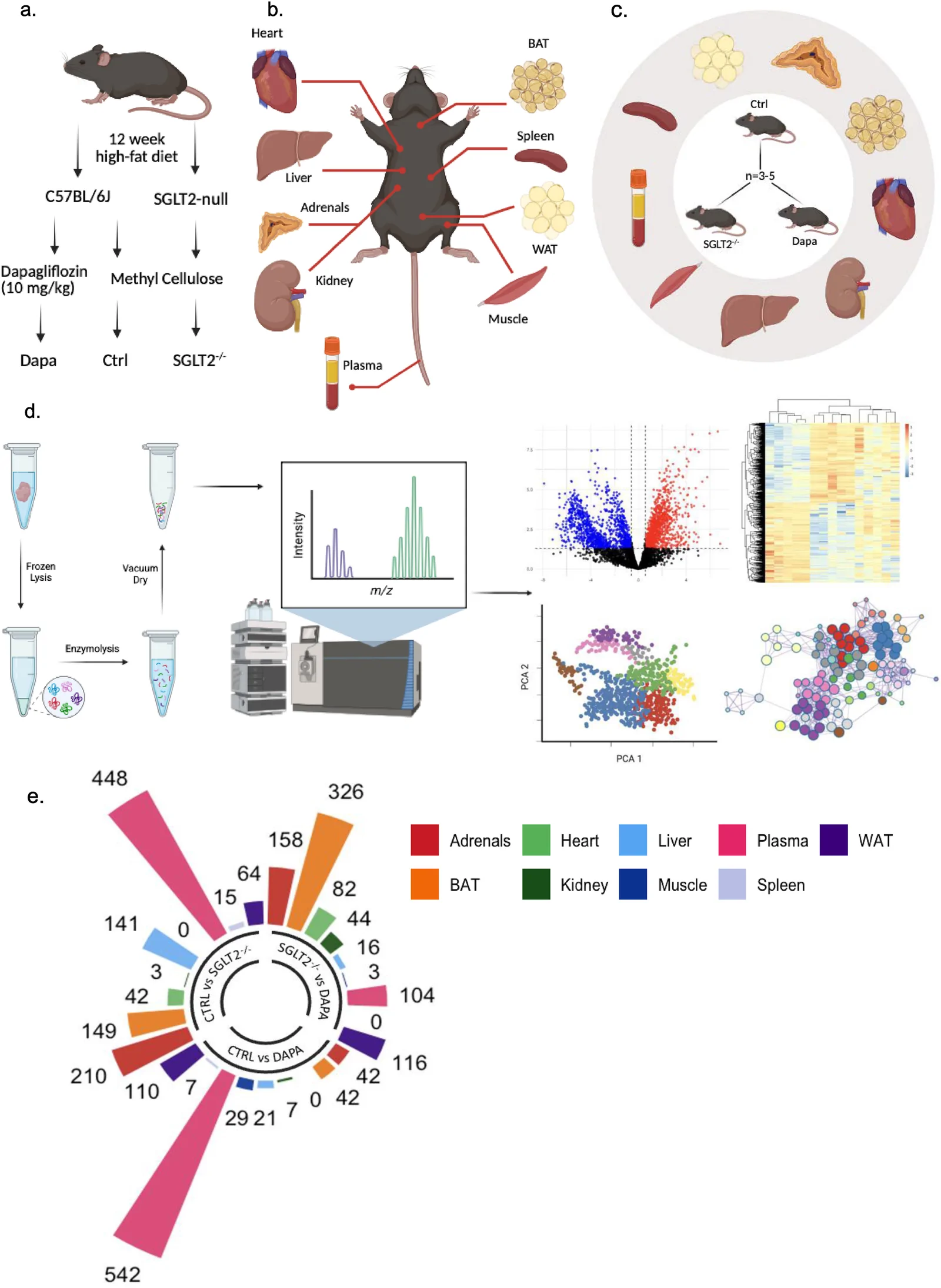
A workflow pipeline of sample collection and bioinformatic analysis. (a) C57BL/6J mice were fed a 12-week high-fat diet (58 kcal% Fat, 18.4% Sucrose) and assigned to one of three experimental conditions: control (Ctrl) left untreated, CRISPR-Cas9-mediated SGLT2 knockout (SGLT2^−/−^), or Ctrl mice treated with 10 mg/kg dapagliflozin (Forxiga; Dapa) for 14 days. (b) Anatomical locations of the eight organs and blood plasma that were selected and harvested for differential proteomic analysis, which include the heart, liver, adrenals, kidney, muscle, white adipose tissue (WAT), spleen and brown adipose tissue (BAT). (c) Summary of the tissues and the number of mice used per experimental group. All experimental conditions (Ctrl, SGLT2^−/−^, Dapa) had 5 of each of the eight organs and plasma besides heart and spleen. (d) Proteomic workflow pipeline and analysis of preparing the various tissues for the liquid chromatography-tandem mass spectrometry (LC-MS/MS), beginning with lysis of frozen issue and enzymolysis to release free peptides, which were freeze dried and passed through the G2S LC-MS/MS for subsequential differential proteomic analysis. (e) A bar plot demonstrating the number of differentially expressed proteins (DEPs) in each of the eight organs and blood plasma per individual comparison (Ctrl vs SGLT2^−/−^, Ctrl vs Dapa, and SGLT2^−/−^ vs Dapa). Organ illustrations were created using BioRender (https://www.biorender.com).

To characterise the molecular consequences of genetic deletion and pharmacological inhibition of SGLT2, we performed functional enrichment analyses on differentially expressed proteins (DEPs) previously identified by Linear Models for Microarray Data (limma) analysis across all harvested tissues [13]. Analyses were conducted separately for each condition relative to control vehicle, SGLT2 null mice (SGLT2^−/−^ vs Control) and dapagliflozin-treated mice (Dapa vs Control), as well as for the comparison between SGLT2 null and dapagliflozin treatment (SGLT2^−/−^ vs Dapa). The comparison between SGLT2^−/−^ and the vehicle control identified 566 DEPs across these eight tissues (Fig. 2a), with 88% of them found in the adrenals (209), BAT (149) and liver (141). The functional enrichment of the DEPs was performed using Gene Ontology (GO) and Kyoto Encyclopedia of Genes and Genomes (KEGG) pathway enrichment analyses, complemented by integrative visualisation and clustering in Metascape [14]. The Metascape Circoplot (Fig. 2b), and the UpSetPlot (Fig. 2c), showed that most of the overlap also occurs among these three organs.

**Fig. 2:**
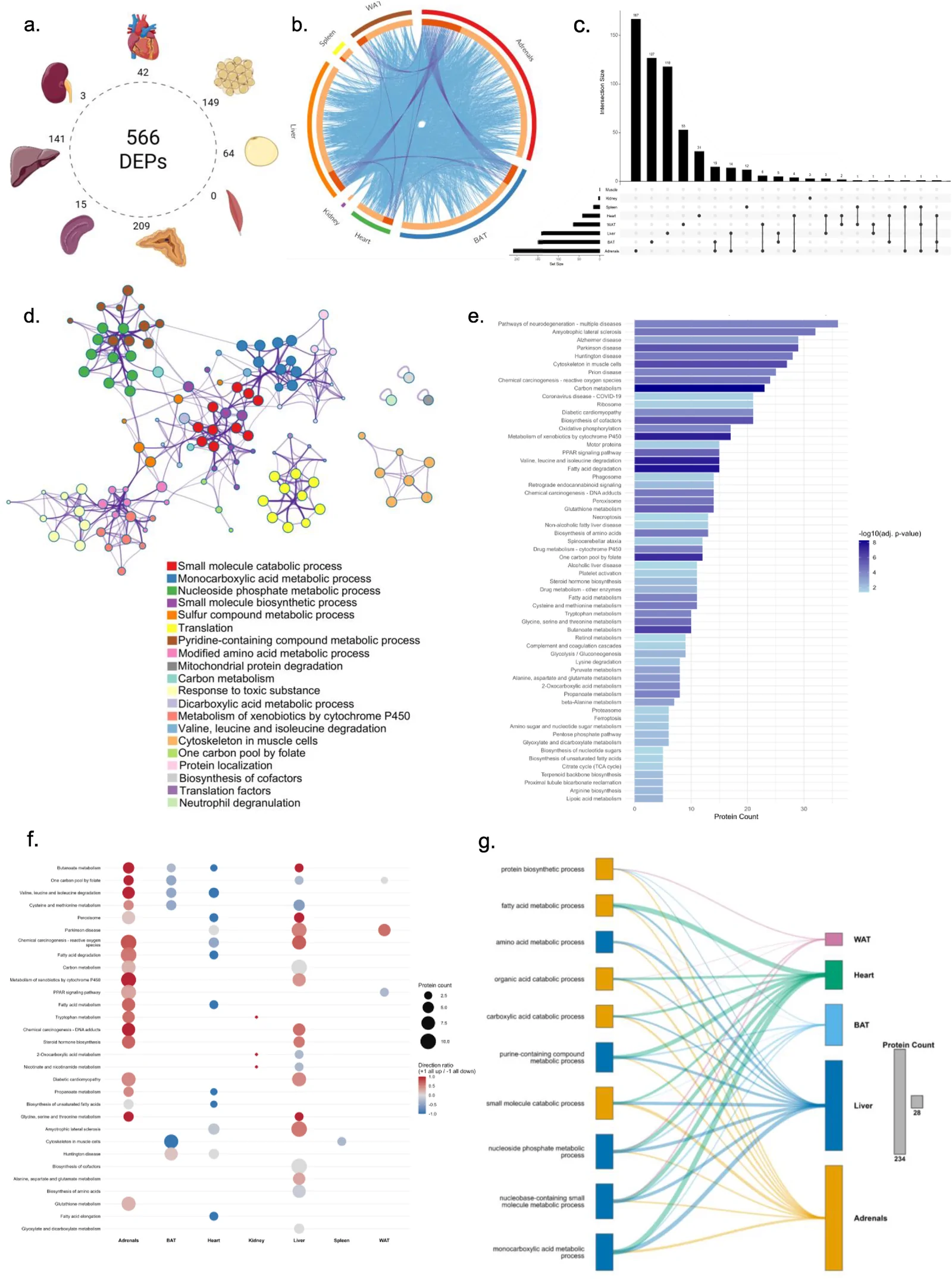
A multi-organ analysis of differentially expressed proteins in SGLT2 null mice (SGLT2^−/−^) versus control. (a) The outer circle represents the total number of differentially expressed proteins (DEPs) per organ. A total of 566 DEPs were identified across all eight organs. Organ illustrations were created using BioRender (https://www.biorender.com) and SMART Servier Medical Art (https://smart.servier.com). (b) The Circos plot demonstrates the inter-organ DEP overlap, with purple links representing shared proteins and blue links representing shared enriched ontology terms. (c) UpSet plot showing at the top bars the number of unique (single black dot) and shared (connected black dots) DEPs across tissues. (d) Enriched ontology clusters of the DEPs were created using Metascape (https://metascape.org) using GO and KEGG ontological pathways, where individual nodes represent proteins that share the same ID cluster and one representative term per cluster is labelled. (e) Enrichment of top 30 KEGG pathways of all 566 DEPs, ordered by number of proteins and coloured based on the -log10 (*p*-value). (f) Top 30 enriched KEGG pathways by tissue, dot size according to protein count and colour representing direction ratio of proteins (red = predominantly upregulated; blue = predominantly downregulated; grey = mixed direction). (g) Top 10 enriched GO Biological Processes by organ. Line width represents the proportion of proteins from each tissue contributing to a given pathway and tissue box height is proportional to its total DEP count. Pathway boxes are coloured according to the colour of the primary tissue contributing to that pathway.

The enrichment ontology clusters from Metascape revealed a primary enrichment for small molecule catabolism, monocarboxylic acid, nucleoside phosphate and carbon metabolic processes with additional smaller clusters related to stress response, cytoskeletal organisation and protein biosynthetic pathways (Fig. 2d). The KEGG enrichment similar metabolic terms were identified, however neurodegeneration-associated pathways dominated the top pathways with higher number of proteins, followed by mitochondrial, lipid, amino acid and carbon metabolism, alongside oxidative stress and detoxification pathways (Fig. 2e). The liver and adrenals show the broadest pathway representation across tissues, with a predominantly upregulated protein expression in various pathways (Fig. 2f). Whilst the heart was enriched in more pathways than BAT despite having fewer DEPs, they both reveal downregulated expression across their enriched pathways. Fatty acid degradation was enriched with high protein abundance and was predominantly upregulated in the adrenals, whereas carbon metabolism and biosynthesis of cofactors showed mixed expression in the liver. Oxidative stress-related terms, notably steroid hormone biosynthesis, peroxisome, chemical carcinogenesis - reactive oxygen species and glutathione metabolism, are also upregulated in the adrenals and liver, besides the final term, which is only enriched in the adrenals. BAT revealed significant downregulation of the cytoskeleton in muscle cells and of branched-chain amino acid metabolic pathways, whilst the heart showed downregulation across fatty acid metabolism, propanoate metabolism and oxidative stress pathways (peroxisome, chemical carcinogenesis). However, from these top 30 KEGG pathways, the kidney and WAT had only three enriched terms, whilst the spleen had one. The Sankey diagram confirms the liver and adrenals as the dominant contributors to GO pathway enrichment, with the heart showing a great percentage of its proteins enriched across these pathways (Fig. 2g). The adrenals exhibited a significant enrichment for energy production and substrate catabolism, whereas the liver showed stronger enrichment in pathways associated with the metabolism of amino acids, nucleotides and other intermediary processes.

### 2.2 Adipose tissues demonstrate heavily dysregulated proteomic expression in dapagliflozin-treated vs. control mice

Differential proteomic analysis between the dapagliflozin-treated versus control mice revealed a total of 556 DEPs across eight tissues, with the highest frequency of DEPs identified in the BAT (386) and WAT (110) (Fig. 3a). Most of the inter-tissue DEP overlap occurred primarily between the adipose tissues, in addition to the adrenals, as depicted in the Circo and Upset plot (Fig. 3b and c). The Metascape functional enrichment protein-protein cluster analysis also revealed significantly enriched core metabolic pathways, such as purine-containing metabolic compounds, GTP hydrolysis and monocarboxylic metabolic processes (Fig. 3d). KEGG pathway enrichment analyses revealed several neurodegenerative diseases that were also identified in the previous comparison with SGLT2^−/−^ mice. Additionally, many oxidative stress-related pathways were also highlighted, including oxidative phosphorylation, fatty acid degradation and glycerolipid metabolism (Fig. 3e). Whilst the tissue-specific enrichment of KEGG pathways revealed many significant pathways highlighted in the BAT and WAT, there was also a notably high frequency of enriched pathways for the muscle. These terms were consistent in highlighting neuropathic diseases and oxidative stress-related terms, with a general downregulatory pattern of expression (Fig. 3f). The GO pathway analysis corroborates the KEGG enrichment, demonstrating that the most dysregulation occurs in metabolic processes within the aforementioned tissues, as well as in the liver (Fig. 3g).

**Fig. 3:**
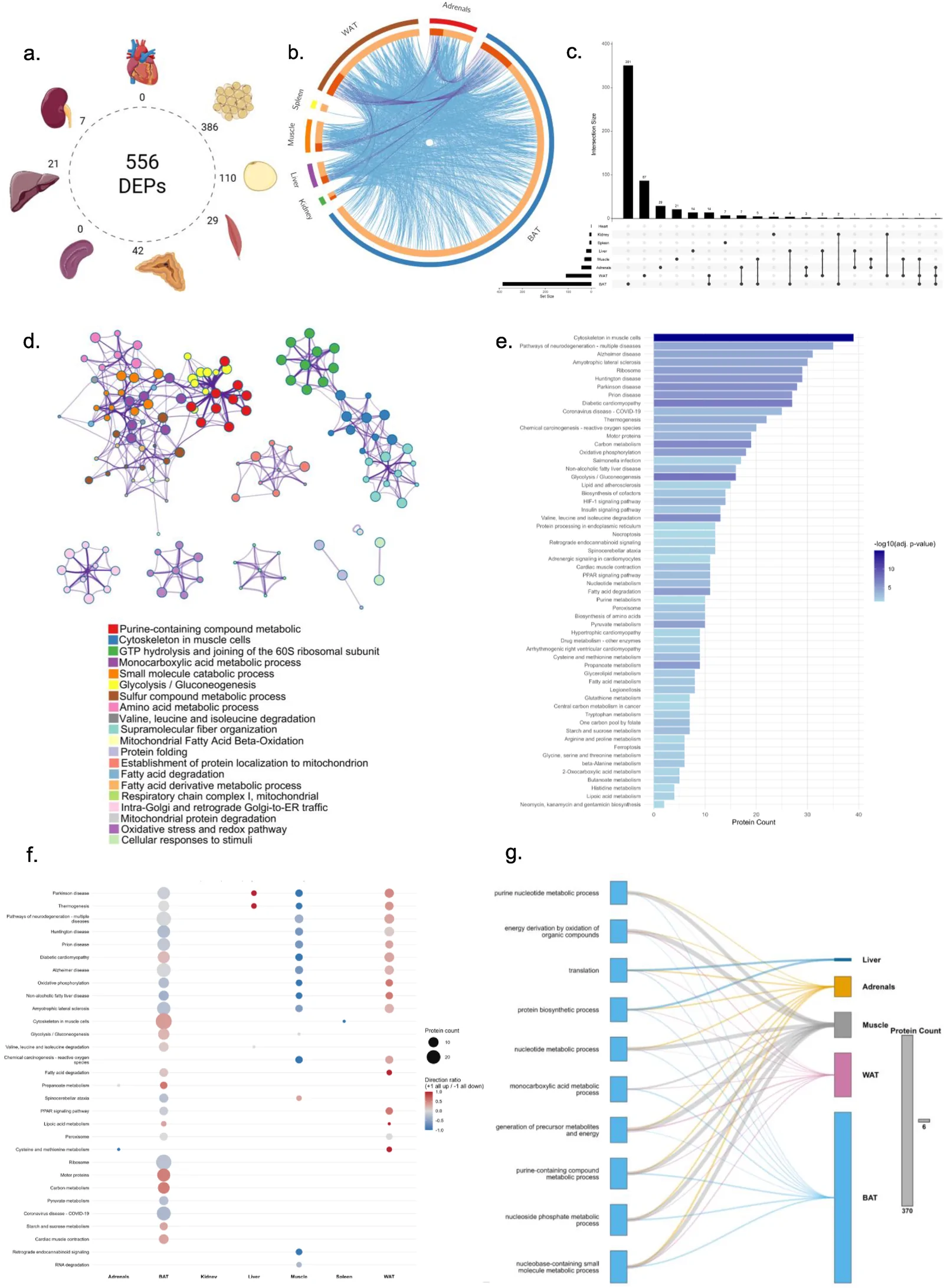
A multi-organ analysis of differentially expressed proteins in dapagliflozin-treated versus control mice. (a) The outer circle represents the total number of DEPs per organ. A total of 556 DEPs were identified across all eight organs. Organ illustrations were created using BioRender (https://www.biorender.com) and SMART Servier Medical Art (https://smart.servier.com). (b) The Circos plot demonstrates the inter-organ DEP overlap, with purple links representing shared proteins and blue links representing shared enriched ontology terms (c) UpSet plot showing at the top bars the number of unique (single black dot) and shared (connected black dots) DEPs across tissues. (d) Enriched ontology clusters of the DEPs were created using Metascape (https://metascape.org) using GO and KEGG ontological pathways, where individual nodes represent proteins that share the same ID cluster and one representative term per cluster is labelled. (e) Enrichment of top 30 KEGG pathways of all 556 DEPs, ordered by number of proteins and coloured based on the -log10 (*p*-value), (f) Top 30 enriched KEGG pathways by tissue, dot size according to protein count and colour representing direction ratio of proteins (red = predominantly upregulated; blue = predominantly downregulated; grey = mixed direction). (g) Top 10 enriched GO Biological Processes by organ. Line width represents the proportion of proteins from each tissue contributing to a given pathway and tissue box height is proportional to its total DEP count. Pathway boxes are coloured according to the colour of the primary tissue contributing to that pathway.

### 2.3 Genetic and pharmacological SGLT2 inhibition show divergent tissue responses

The proteomic analysis comparing genetic and pharmacological inhibition of SGLT2 revealed a higher number of DEPs (668) compared to the individual comparisons with the controls. BAT (326), adrenals (158) and WAT (116) are highlighted as before, whereas kidney (44) and heart (82) show the biggest increase in DEPs yet (Fig. 4a). The Circoplot and UpSet plot showed the inter-tissue overlap concentrated within these dominant tissues (Fig. 4b and c). Metascape enrichment ontology clusters revealed similar processes to the individual comparisons (Fig. 4d). KEGG enrichment mirrored prior findings with neurodegeneration associated pathways accounting for the top of protein counts followed by cytoskeleton in muscle cells, chemical carcinogenesis, diabetic cardiomyopathy, thermogenesis and oxidative phosphorylation (Fig. 4e). In the organ KEGG pathway analysis, adrenals, kidney and liver showed mainly upregulation with a few neutral terms, whereas BAT displayed a predominant downregulation, heart only neutral terms and WAT a mixed pattern (Fig. 4f). Adrenals were enriched mainly in metabolic terms, while liver had upregulation in oxidative stress terms alongside non-alcoholic fatty liver disease which was also enriched in the other tissues except adrenals. Kidney was enriched in a few pathways related to one-carbon metabolism. In contrast, BAT had the highest protein counts, with cytoskeletal and central carbon metabolism downregulated and thermogenesis and PPAR signalling pathway upregulated. Moreover, oxidative phosphorylation is enriched for the first time in five tissues simultaneously, upregulated in the kidney, liver and BAT, but slightly downregulated in WAT and neutral in the heart. The Sankey diagram confirms BAT and adrenals with the higher number of proteins for the GO pathways, but liver, kidney and heart show a significant proportion of their DEPs enriched across these pathways, which centre on nucleotide metabolism, substrate catabolism, and energy production (Fig. 4g).

**Fig. 4:**
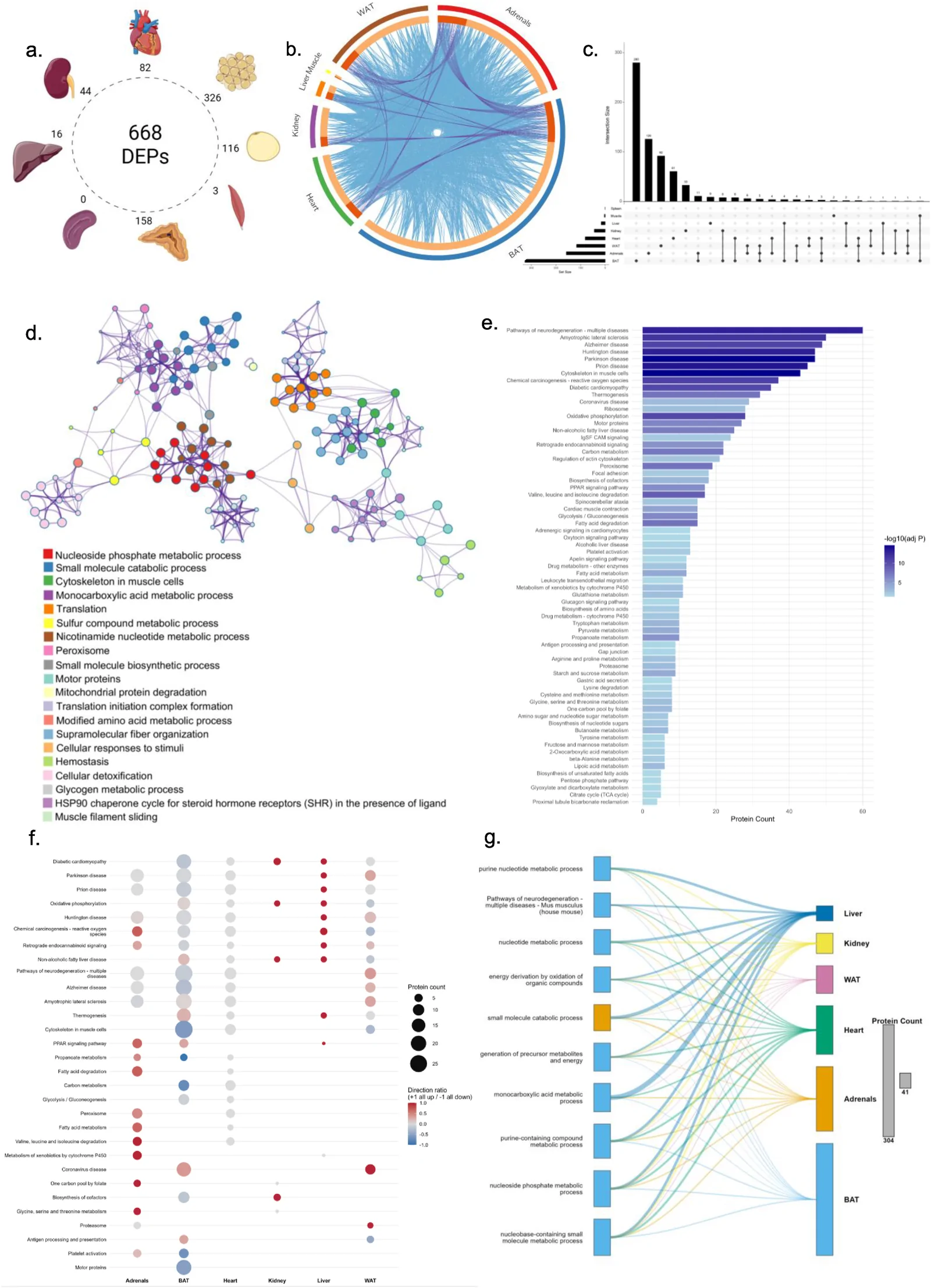
A multi-organ analysis of differentially expressed proteins in SGLT2 null mice (SGLT2^−/−^) versus dapagliflozin-treated mice. (a) The outer circle represents the total number of differentially expressed proteins (DEPs) per organ. A total of 668 DEPs were identified across all eight organs. Organ illustrations were created using BioRender (https://www.biorender.com) and SMART Servier Medical Art (https://smart.servier.com). (b) The Circos plot demonstrates the inter-organ DEP overlap, with purple links representing shared proteins and blue links representing shared enriched ontology terms. (c) UpSet plot showing at the top bars the number of unique (single black dot) and shared (connected black dots) DEPs across tissues. (d) Enriched ontology clusters of the DEPs were created using Metascape (https://metascape.org) using GO and KEGG ontological pathways, where individual nodes represent proteins that share the same ID cluster and one representative term per cluster is labelled. (e) Enrichment of top 30 KEGG pathways of all 668 DEPs, ordered by number of proteins and coloured based on the -log10 (*p*-value). (f) Top 30 enriched KEGG pathways by tissue, dot size according to protein count and colour representing direction ratio of proteins (red = predominantly upregulated; blue = predominantly downregulated; grey = mixed direction). (g) Top 10 enriched GO Biological Processes by organ. Line width represents the proportion of proteins from each tissue contributing to a given pathway and tissue box height is proportional to its total DEP count. Pathway boxes are coloured according to the colour of the primary tissue contributing to that pathway.

### 2.4 Overlap analysis reveals a common oxidative stress signature in DEPs shared between genetic and pharmacological SGLT2 inhibition in multi-organ proteomics

Overlaps of DEPs and pathways between SGLT2^−/−^ vs Control and Dapa vs Control were analysed to investigate common DEPs and pathways between genetic and pharmacological SGLT2 inhibition. Both comparisons share 214 DEPs while 352 and 342 proteins were unique to SGLT2^−/−^ and Dapa respectively (Fig. 5a). Amongst the 214 common DEPs, 85 proteins (39.7%%) are related to oxidative stress and ROS response, while the remaining 129 (60.3%) are involved in other biological processes, indicating that almost 40% of the shared proteins are annotated in at least one oxidative stress-related pathways (Fig. 5b). Oxidative stress-related GO terms were defined based on a broad curated list encompassing core ROS response, antioxidant defence, mitochondrial ROS, lipid oxidation, DNA damage, and ER stress pathways (Supplementary Table 1). At the pathway level, GO enrichment analysis of all DEPs, identified 337 significant pathways shared between both comparisons (Fig. 5c). GO enrichment of oxidative stress biological processes among the shared proteins identified significant enrichment of aerobic respiration, accounting for the highest protein count and significance followed by response to oxidative stress, detoxification terms and multiple mitochondrial pathways focused on oxidative phosphorylation, as well as glutathione metabolism (Fig. 5d). The high enrichment of oxidative stress and ROS-related pathways in the common DEPs prompted our focus on oxidative stress in the following sections.

**Fig. 5:**
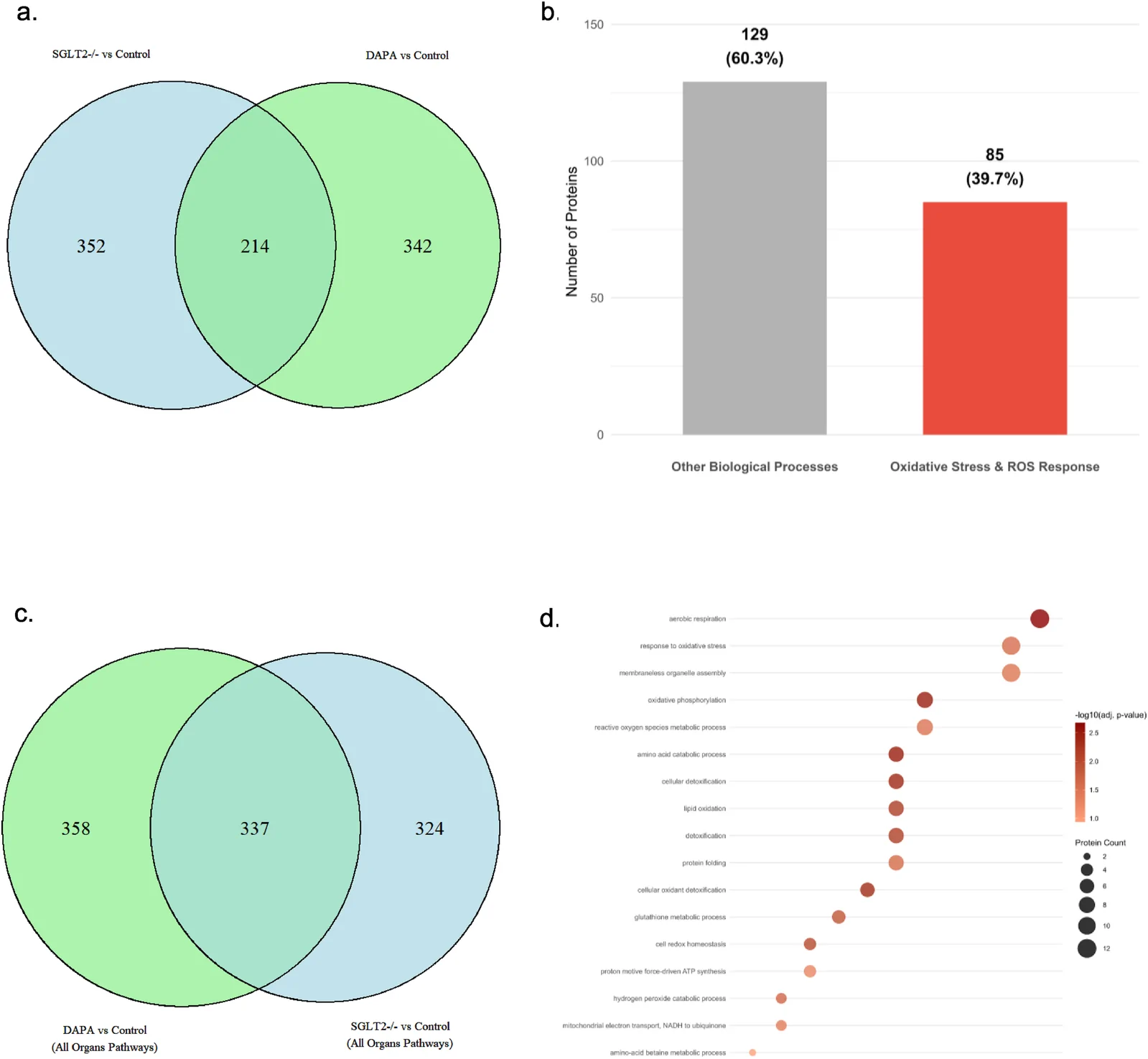
Common differentially expressed proteins, oxidative stress enrichment, and shared pathways between SGLT2−/− vs Control and Dapa vs Control across all tissues. (a) Venn diagram of common 214 differentially expressed proteins including (b) 85 (39.7%) oxidative stress proteins identified by direct annotation to curated GO terms related to ROS metabolism, antioxidant defence, oxidative phosphorylation and related processes in the common DEPs. (c) Venn diagram of significant GO biological processes in SGLT2−/− vs Control and Dapa vs Control. The intersection shows 337 commonly enriched pathways between comparisons. (d) Gene Ontology enrichment analysis of significant oxidative stress related pathways. Dot size indicates protein count and colour represents -log10(q-value).

### 2.5 Liver and adrenals continue as the organs with the most pronounced oxidative stress-specific proteomic dysregulation

The oxidative stress-specific proteomic sub-analysis between SGLT2-null and control mice revealed a total of 219 DEPs related to oxidative stress. Much of the oxidative-specific protein dysregulation occurred within the adrenals (79), liver (74) and BAT (49), with the highest inter-organ DEP overlap occurring within these organs, but also in the heart (Fig. 6a and b). Metascape's integrative cluster analysis of protein-protein interaction revealed six key processes across all oxidative stress-related DEPs. Most notably, a large proportion of DEPs (54.1%) were highlighted under the term “Chemical carcinogenesis - Reactive Oxygen Species' (Fig. 6c). Our focus on oxidative stress is further supported by the organ-specific GO pathway analysis, highlighting key oxidative stress terms such as glutathione synthesis, mitochondrial ATP transport, and NADH to ubiquinone transport across multiple organs, which are generally upregulated in the adrenals and liver, but downregulated in the heart (Fig. 6d). The relative DEP frequencies per oxidative stress-related GO biological process is highlighted in the Sankey diagram, demonstrating the adrenals and liver contributing to the dysregulation of many of these processes in the absence of SGLT2 (Fig. 6e). The expression profile of some of the most significant oxidative stress-related DEPs in the heatmap demonstrates upregulated (red) and downregulated (blue) proteins across various organs, grouped by similar expression profiles. The highest levels of dysregulation among these select DEPs were most prominent in the BAT, liver and adrenals, demonstrating a general trend towards the upregulation of proteins (Fig. 6f).

**Fig. 6:**
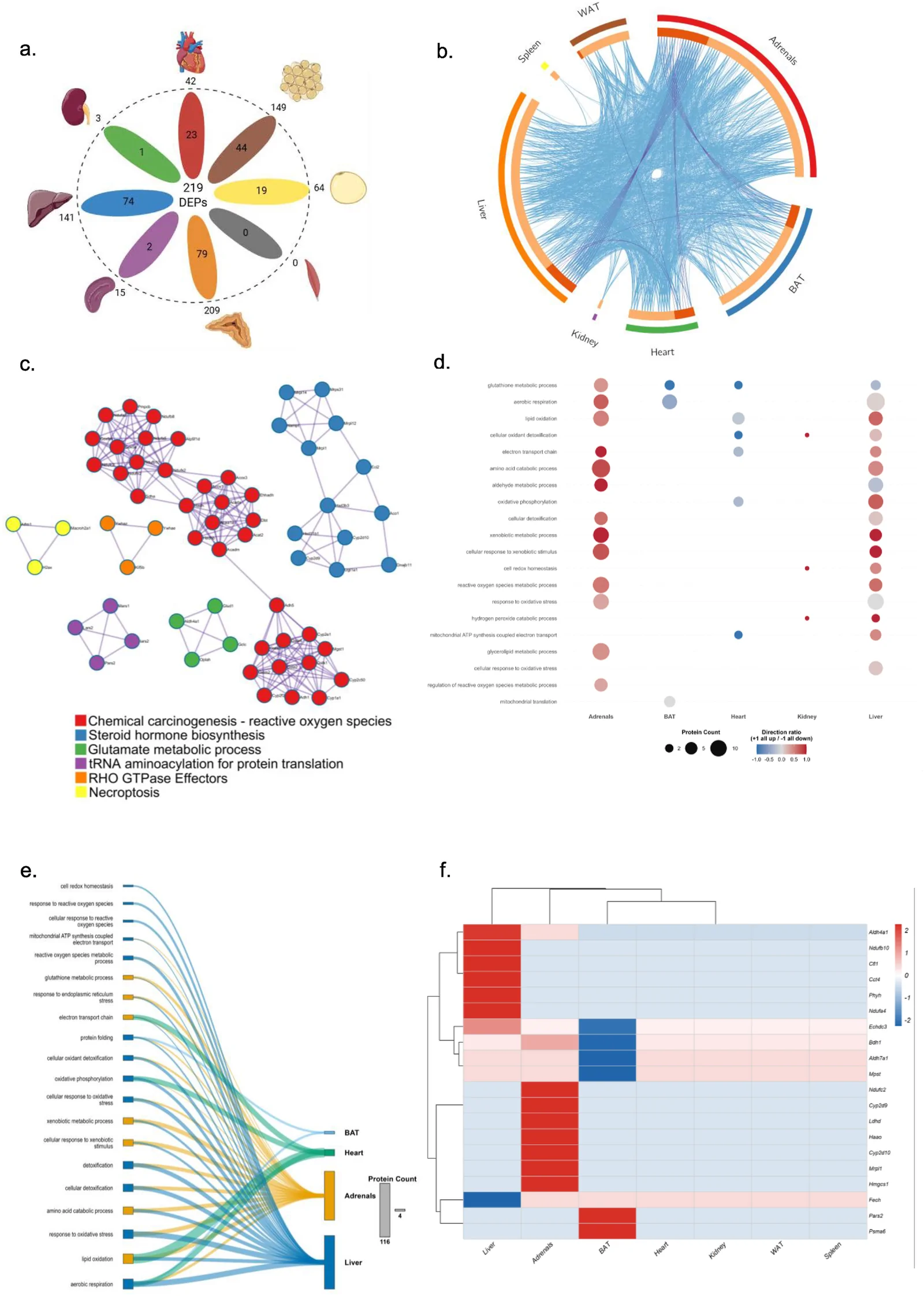
Oxidative stress-related differentially expressed proteins across eight organs in SGLT2-null versus control mice. (a) The outer circle represents the total number of differentially expressed proteins (DEPs) per organ, whilst the inner circle denotes those related to oxidative stress. A total of 220 oxidative stress-related DEPs were identified between SGLT2^−/−^ and control mice. Organ illustrations were created using BioRender (https://www.biorender.com) and SMART Servier Medical Art (https://smart.servier.com). (b) The Circo plot demonstrates the inter-organ oxidative stress DEP overlap, with purple links representing identical proteins and blue links representing closely related proteins. (c) Enrichment clusters of the DEPs were created using Metascape (https://metascape.org) using GO and KEGG ontological pathways, where individual nodes represent proteins that share the same ID cluster. (d) Enrichment of KEGG pathways of all 220 oxidative stress-related DEPs, ordered by significance and coloured based on the direction of change; upregulated pathways are coloued in red and downregulated pathways are coloured blue. Pathways have been separated by organ, with the size of the dot representing the protein counts contained in the annotation. (e) GO biological processes organised by organ, with the height of the bar on the right and the width of the lines connecting organs to GO annotations represent protein counts. (f) A heatmap of the most significantly differentially expressed proteins by organ, with red representing upregulation and blue representing downregulation of the protein.

### 2.6 Adipose tissues maintain their extensive protein dysregulation in oxidative stress-specific processes

A similar oxidative stress-specific analysis comparing dapagliflozin-treated and control mice revealed a total of 209 DEPs related to oxidative stress, with the majority found in the adipose tissues (BAT = 148, WAT = 35). There was also a notable frequency of DEPs highlighted in the muscle and adrenals, which overlapped with DEPs found in the adipose tissues (Fig. 7a and b). The Metascape protein-protein interaction clusters revealed six different, but still oxidative stress-related, pathways where the term ‘Oxidative stress’ harboured the most mapped DEPs (39.7%) (Fig. 7c). Organ-specific GO pathway analysis revealed key oxidative stress terms across the adipose tissues, liver and muscle, including oxidative phosphorylation, electron transport chain, and aerobic respiration; all key pathways involved in producing forms of oxidative stress have a general trend for downregulation across organs (Fig. 7d). Protein counts were distributed across the top 20 oxidative stress GO pathway terms by organ, which showed heavy involvement of the liver in oxidative stress and reactive oxygen species (ROS), whilst DEPs from other organs were more evenly distributed across the oxidative GO terms (Fig. 7e). The expression profile of the most significant oxidative stress-related DEPs across organs demonstrating upregulated (red) and downregulated (blue) proteins show a much larger spread of affected organs than that of the SGLT2-null mice but also show a trend towards protein downregulation (Fig. 7f).

**Fig. 7:**
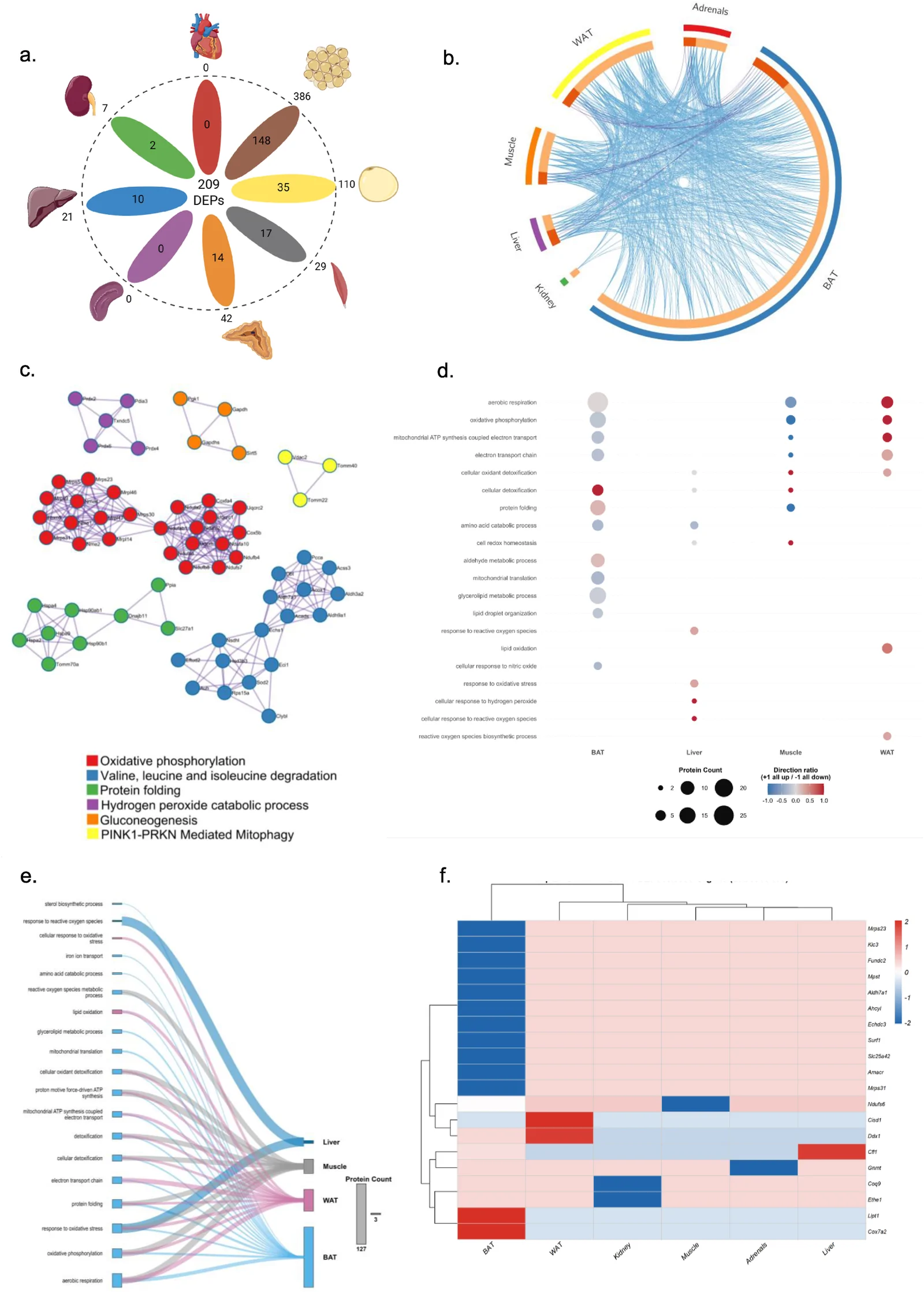
Oxidative stress-related differentially expressed proteins across eight organs in dapagliflozin-treated versus control mice. (a) The outer circle represents the total number of differentially expressed proteins (DEPs) per organ, whilst the inner circle denotes those related to oxidative stress. A total of 211 oxidative stress-related DEPs were identified between dapagliflozin-treated and control mice. Organ illustrations were created using BioRender (https://www.biorender.com) and SMART Servier Medical Art (https://smart.servier.com). (b) The Circo plot demonstrates the inter-organ oxidative stress DEP overlap, with purple links representing identical proteins and blue links representing closely related proteins. (c) Enrichment clusters of the DEPs were created using Metascape (https://metascape.org) using GO and KEGG ontological pathways, where individual nodes represent proteins that share the same ID cluster. (d) Enrichment of KEGG pathways of all 211 oxidative stress-related DEPs, ordered by significance and coloured based on the direction of change; upregulated pathways are coloured in red and downregulated pathways are coloured blue. Pathways have been separated by organ, with the size of the dot representing the protein counts contained in the annotation. (e) GO biological processes organised by organ, with the height of the bar on the right and the width of the lines connecting organs to GO annotations represent protein counts. (f) A heatmap of the most significantly differentially expressed proteins by organ, with red representing upregulation and blue representing downregulation of the protein.

### 2.7 Kidney emerges as a novel contributor to oxidative stress dysregulation in the comparison of genetic and pharmacological SGLT2 inhibition

The oxidative stress proteomic sub-analysis between SGLT2^−/−^ and Dapa revealed 252 DEPs (Fig. 8a) and, as in the previous comparison, BAT (123) remained the dominant tissue, followed by adrenals (58), WAT (37). Metascape's integrative cluster analysis identified ten key processes, including the electron transport chain and fatty acid degradation (Fig. 8c). In the organ GO pathway analysis, adrenals, kidney, liver and muscle showed upregulation across their enriched terms. In contrast, the heart and WAT showed downregulation, with a mixed pattern of regulation shown in the BAT (Fig. 8d). Kidney, liver and muscle enrichment was largely restricted to mitochondrial respiratory terms, while the adrenals additionally had glutathione, xenobiotic and amino acid metabolism, as well as lipid oxidation. BAT had the highest frequency of enriched terms with respiratory chain terms neutral or slightly upregulated, whereas detoxification terms were downregulated. Notably, the kidney emerged for the first time as a strongly enriched tissue, as further reflected in Fig. 8e, where the thickest lines correspond to its connections to aerobic respiration and oxidative phosphorylation-related terms, indicating that its oxidative stress-related DEPs are almost entirely concentrated within mitochondrial respiratory processes. The expression profiles of the most significant oxidative stress proteins were slightly downregulated across all tissues, with the exception of greater dysregulation predominantly in adipose tissues and the adrenals (Fig. 8f).

**Fig. 8:**
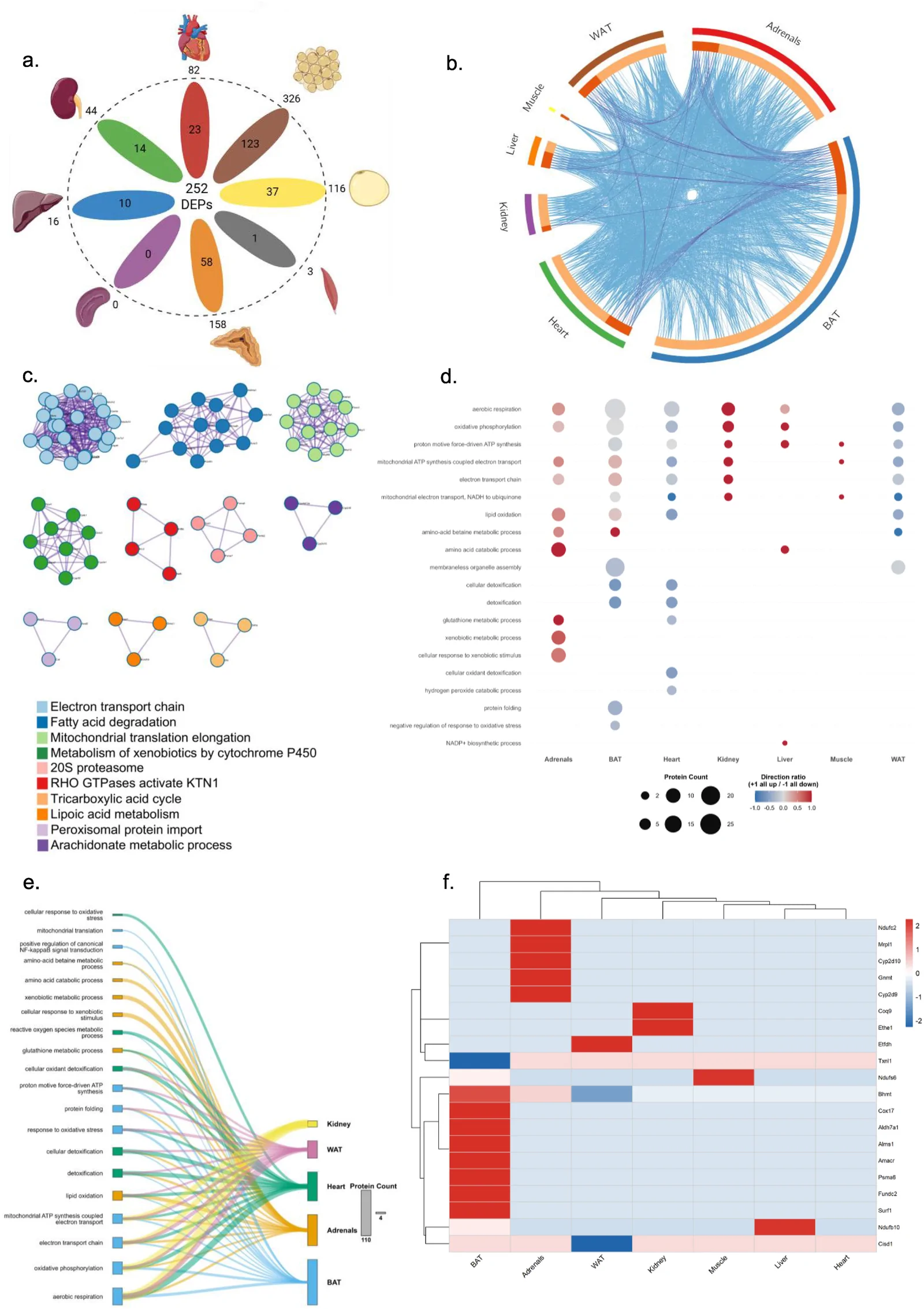
Oxidative stress-related differentially expressed proteins across eight organs in SGLT2-null versus dapagliflozin-treated mice. (a) The outer circle represents the total number of differentially expressed proteins (DEPs) per organ, whilst the inner circle denotes those related to oxidative stress. A total of 252 oxidative stress-related DEPs were identified between dapagliflozin-treated and control mice. Organ illustrations were created using BioRender (https://www.biorender.com) and SMART Servier Medical Art (https://smart.servier.com). (b) The Circo plot demonstrates the inter-organ oxidative stress DEP overlap, with purple links representing identical proteins and blue links representing closely related proteins. (c) Enrichment clusters of the DEPs were created using Metascape (https://metascape.org) using GO and KEGG ontological pathways, where individual nodes represent proteins that share the same ID cluster. (d) Enrichment of KEGG pathways of all 252 oxidative stress-related DEPs, ordered by significance and coloured based on the direction of change; upregulated pathways are coloured in red and downregulated pathways are coloured blue. Pathways have been separated by organ, with the size of the dot representing the protein counts contained in the annotation. (e) GO biological processes organised by organ, with the height of the bar on the right and the width of the lines connecting organs to GO annotations represent protein counts. (f) A heatmap of the most significantly differentially expressed proteins by organ, with red representing upregulation and blue representing downregulation of the protein.

### 2.8 Plasma differentially expressed proteins functional enrichment analysis

We previously reported the plasma analysis of the three mouse groups [15]. We re-analysed the proteomics dataset in accordance with the script utilised for the tissue analysis, omitting the SPECU, RUViii, and PRPS batch-correction steps, which were omitted due to the lack of QC samples in the plasma dataset. Principal component analysis revealed a clear difference between all groups, especially in control mice (Fig. 9a). We quantified a total of 5660 proteins. In SGLT2 null mice, 318 proteins were significantly upregulated, and 130 downregulated, compared to vehicle controls (Fig. 9b), while in dapagliflozin-treated mice, 168 of the proteins were downregulated and 374 were upregulated (Fig. 9c). SGLT2 null mice and dapagliflozin-treated mice yielded considerably fewer DEPs, 44 significantly upregulated and 60 downregulated (Fig. 9d). Pathway and protein-protein interaction analysis were performed through Metascape as described above. In both SGLT2-null and dapagliflozin-treated mice compared to the controls, enriched ontology cluster analyses confirmed our previous findings that the DEP functions were primarily clustered in the biological process of small-molecule catabolism, as well as sulfur metabolism, response to stilbenoids, and the PPARγ signalling pathway (Fig. 9d and e). In contrast, the comparison between SGLT2 genetic and pharmacological inhibition shifted away from metabolic processes, clustering predominantly around haemostatic and coagulation related processes (Fig. 9f). Of note, the comparisons with the controls demonstrated significant upregulation in proteins involved in fatty acid metabolism and degradation, while with each other only enriched five terms mostly downregulated including TCA cycle, carbon and amino acid metabolism pathways (Fig. 9f).

**Fig. 9:**
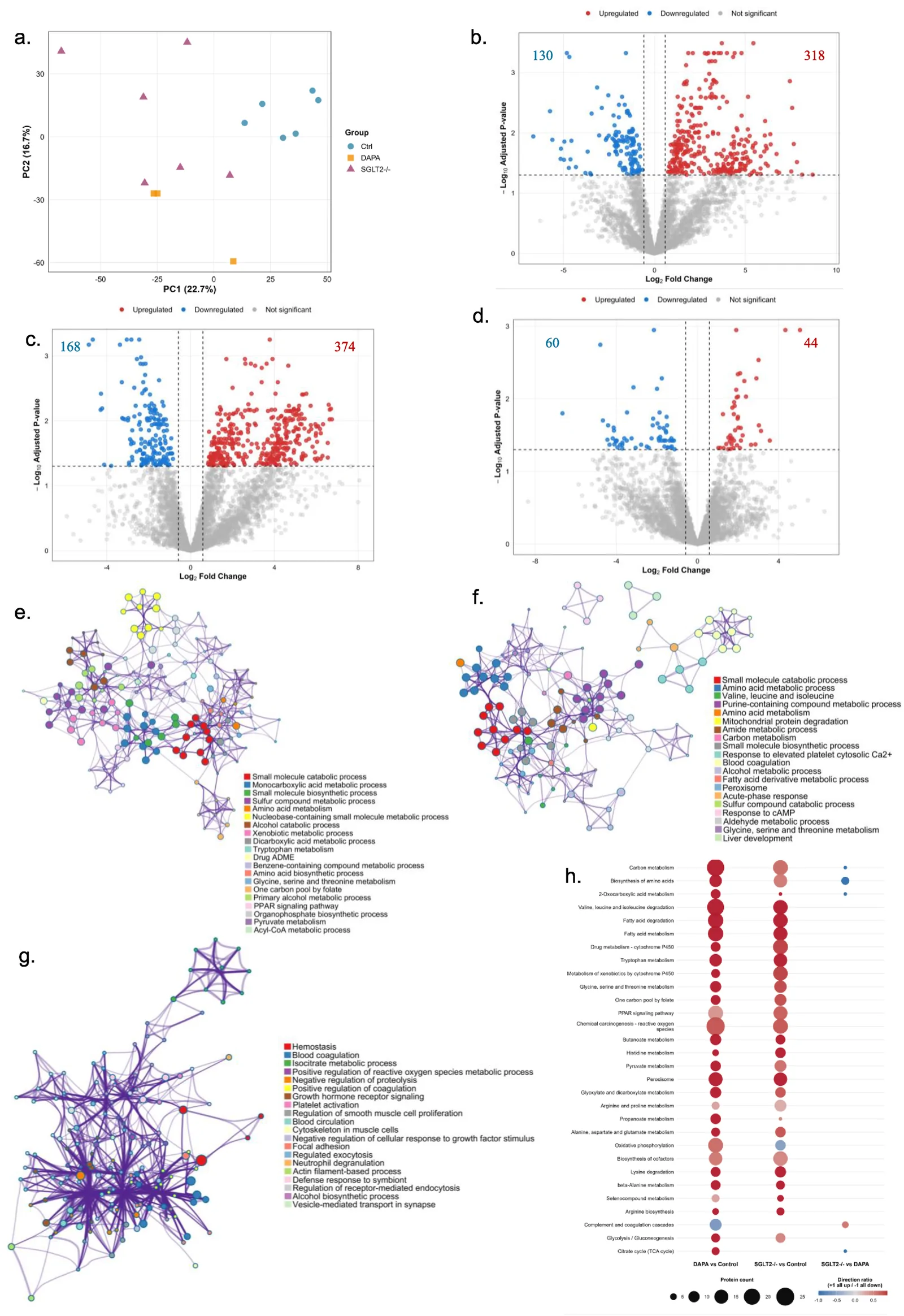
Plasma differentially expressed proteins analysis of SGLT2−/− vs Control and Dapa vs Control across all tissues. (a) Principal Component Analysis (PCA) plot of plasma proteomics after ARSyNseq normalisation. Each point represents one biological replicate, coloured and shaped by experimental group: Ctrl (blue circle), SGLT2^−/−^ (purple triangle) Dapa (orange square). PC1 and PC2 explain 22.7% and 16.7% of total variance respectively. Volcano plot of (b) SGLT2^−/−^ vs Control and (c) Dapa vs Control with significantly upregulated DEPs highlighted in red and those downregulated are highlighted in blue, with significance thresholds defined as p < -log10 (0.05) and log2 fold change > (1.5). Enrichment clusters of DEPs were created using Metascape using GO and KEGG ontological pathways for (d) SGLT2^−/−^ vs Control (e) Dapa vs Control, where individual nodes represent proteins that share the same ID cluster. (f) Top 30 enriched KEGG pathways of SGLT2−/− versus Control and Dapa vs Control. Dot size represents the number of DEPs associated with each pathway (protein count). Dot colour indicates direction ratio where red indicates predominantly upregulated proteins, blue indicates predominantly downregulated proteins, and grey indicates a mixed directionality. Pathways with raw p-value < 0.01 (Metascape standard) are shown.

### 2.9 Genetic and pharmacological SGLT2 inhibition reveal a conserved plasma oxidative stress signature with enhanced mitochondrial respiration in dapagliflozin-treated mice

Following the same approach applied to the other tissues, overlapping analysis in plasma identified 163 DEPs (Fig. 10a) and 316 significant GO biological processes (Fig. 10c) common to SGLT2 null and dapagliflozin-treated mice, both relative to vehicle controls. From the shared DEPs, 84 proteins (51.5%) were mapped to oxidative stress GO terms, while the remaining 79 (48.5%) were mapped to other biological processes, indicating that more than half of the shared plasma proteins are annotated to oxidative stress-related pathways (Fig. 10b). Among the oxidative stress-specific GO Biological Processes terms enriched in the shared plasma proteins, amino acid and lipid oxidation showed higher significance and protein counts. However, glutathione metabolism and response to oxidative stress and ROS are also shown in the multi-organ analysis (Fig. 8d).

**Fig. 10:**
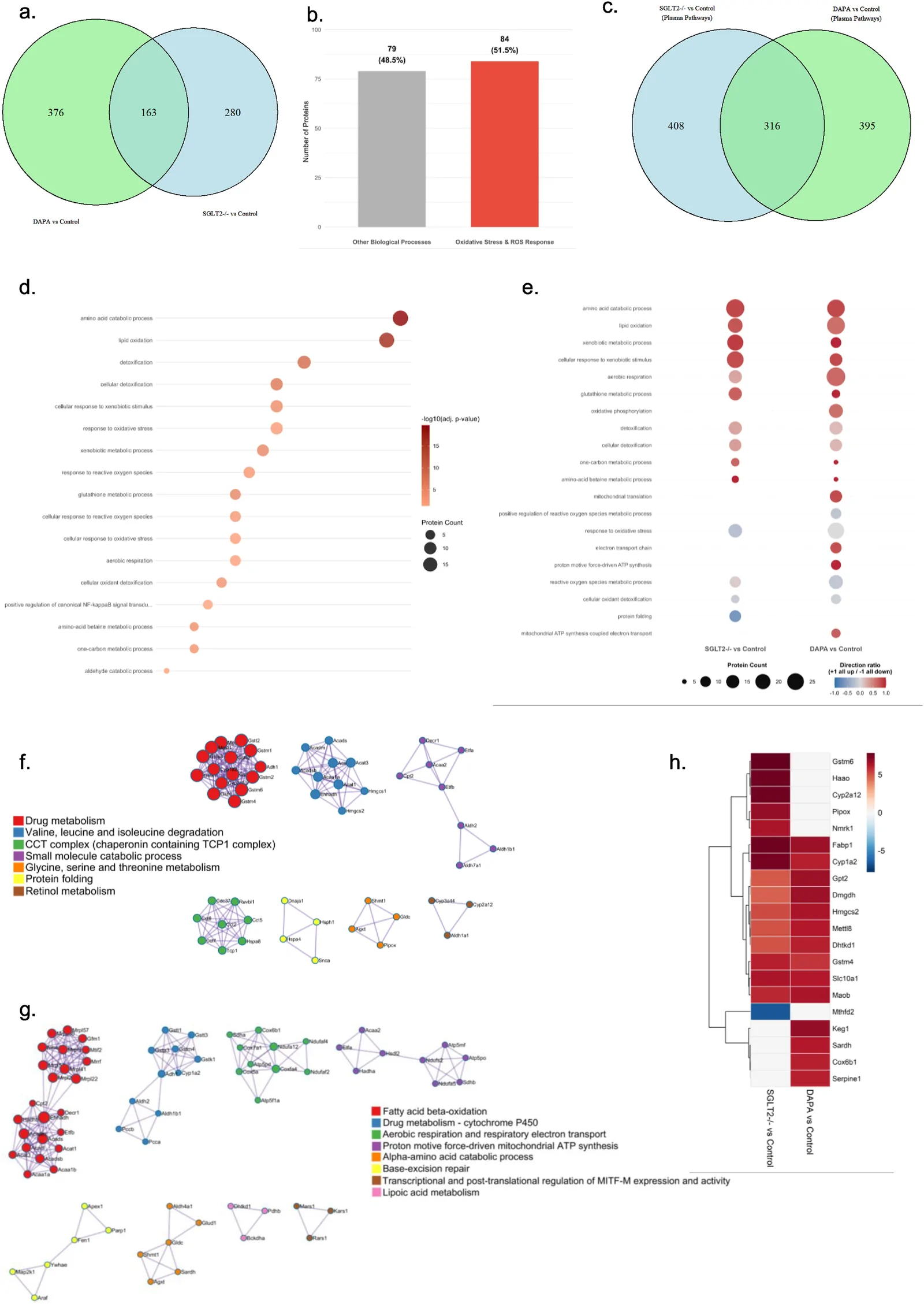
Common differentially expressed proteins and pathways, oxidative stress enrichment in SGLT2−/− vs Control and Dapa vs Control in plasma. (a) Venn diagram of common 163 differentially expressed proteins including (b) 84 (51.5%) oxidative stress proteins identified by direct annotation to curated GO terms related to ROS metabolism, antioxidant defence, oxidative phosphorylation and related processes in the common DEPs. (c) Venn diagram of significant GO biological processes in SGLT2−/− vs Control and Dapa vs Control. The intersection shows 316 commonly enriched pathways between comparisons. (d) Gene Ontology enrichment analysis of significant oxidative stress related pathways. Dot size indicates protein count and colour represents -log10(q-value). (e) Enrichment of KEGG pathways of all oxidative stress-related DEPs, ordered by significance and coloured based on the direction of change; upregulated pathways are coloured in red and downregulated pathways are coloured blue. Pathways have been separated by group comparison, with the size of the dot representing the protein counts contained in the annotation. (f) Enrichment clusters of the DEPs in SGLT2−/− vs. control mice were created using Metascape (https://metascape.org) using GO and KEGG ontological pathways, where individual nodes represent proteins that share the same ID cluster (g) Enrichment clusters of the DEPs in dapagliflozin-treated vs. control mice were created using Metascape using GO and KEGG ontological pathways, where individual nodes represent proteins that share the same ID cluster (h) A heatmap of the most significantly differentially expressed proteins by group comparison, with red representing upregulation and blue representing downregulation of the protein.

The oxidative stress plasma analysis did not include a SGLT2^−/−^ versus dapagliflozin comparison, as the 28 DEPs identified were insufficient to support meaningful enrichment analysis, yielding a single Metascape cluster of three proteins and no significantly enriched oxidative stress terms in the dot plot. In plasma, the oxidative stress-related DEPs analysis revealed a common enrichment between the other two comparisons in more than half of the top 20 GO Biological Processes, including amino acid catabolic process, xenobiotic response, detoxification processes, glutathione metabolic process and aerobic respiration (Fig. 10e). Both comparisons show mixed protein expression or even overall downregulation in response to oxidative stress and ROS metabolism. Notably, mitochondrial pathways, particularly related to oxidative phosphorylation, were largely enriched in dapagliflozin-treated mice compared to SGLT2-null mice. Furthermore, protein-protein interaction network analysis shows a drug metabolism cluster in both SGLT2 null (Fig. 10f) and dapagliflozin-treated mice (Fig. 10g). However, in contrast to SGLT2^−/−^, the dapagliflozin-treated mice show a more cohesive network centred around mitochondrial clusters related to fatty acid beta-oxidation, aerobic respiration and oxidative phosphorylation (Fig. 10g). The heatmap of the top 20 oxidative stress DEPs showed shared upregulation of several proteins between comparisons involved in fatty acid and ketogenic metabolism, detoxification and mitochondrial activity, while the most significant expressions belong to Gstm6 and Fabp1 in SGLT2^−/−^ mice (Fig. 10h).

### 2.10 Integrated metabolic module scoring across comparisons

The three comparisons were analysed regarding their overall protein expression in glycolysis/gluconeogenesis, pyruvate metabolism, TCA cycle, fatty acid degradation, ketone metabolism and OXPHOS, as well as selected individual proteins in the dominant tissues and plasma, yielding two heatmaps (Fig. 11a and b). In SGLT2^−/−^ versus control, liver and adrenals showed upregulation of oxidative phosphorylation and fatty acid degradation and downregulation of the TCA cycle, whereas glycolysis and pyruvate metabolism were upregulated in adrenals but down in the liver. BAT was downregulated across all its scored modules (glycolysis, pyruvate and fatty acid degradation). Conversely, the WAT showed selective upregulation of the TCA cycle and oxidative phosphorylation and downregulation of glucose uptake. At the individual protein level, liver and adrenals showed increased ketone, β-oxidation and electron transport chain proteins (Fig. 11b). In dapagliflozin-treated mice versus control, WAT was upregulated across all modules and strongly for fatty acid degradation, BAT showed reduced expression of pyruvate metabolism and fatty acid degradation alongside increased expression of TCA cycle and multiple glycolysis/gluconeogenesis proteins, and muscle displayed marked downregulation of OXPHOS. In the SGLT2^−/−^ versus dapagliflozin comparison, the kidney downregulated glucose uptake and upregulation of glycolysis/gluconeogenesis and oxidative phosphorylation, which was also significantly upregulated in the muscle, in contrast to its reduction in dapagliflozin versus control. The heart scored only in this comparison, as did the kidney, with a reduction in pyruvate metabolism, the TCA cycle and fatty acid degradation. Adipose tissues diverged, with BAT upregulating pyruvate metabolism and fatty acid degradation, whilst WAT was downregulated across all modules. Plasma was upregulated across nearly all modules in both control comparisons, but in none for the comparison between inhibition and deletion of SGLT2. The spleen showed no changes in any comparison, and ketone metabolism was never scored in any tissues or comparison. Given the canonical role of SGLT2 in renal glucose reabsorption, we additionally examined glucose transporter abundance across the tissue proteomic datasets. SGLT2/SLC5A2 was identified as differentially expressed only in the kidney, consistent with its predominant renal expression. No significant differential abundance of SGLT1/SLC5A1, GLUT1/SLC2A1 or GLUT4/SLC2A4 was detected across the tissue datasets. Together with the tissue-specific changes in the glucose-uptake-associated and downstream metabolic modules shown in Fig. 11, these findings indicate metabolic remodelling without widespread changes in the abundance of major glucose transporters.

**Fig. 11:**
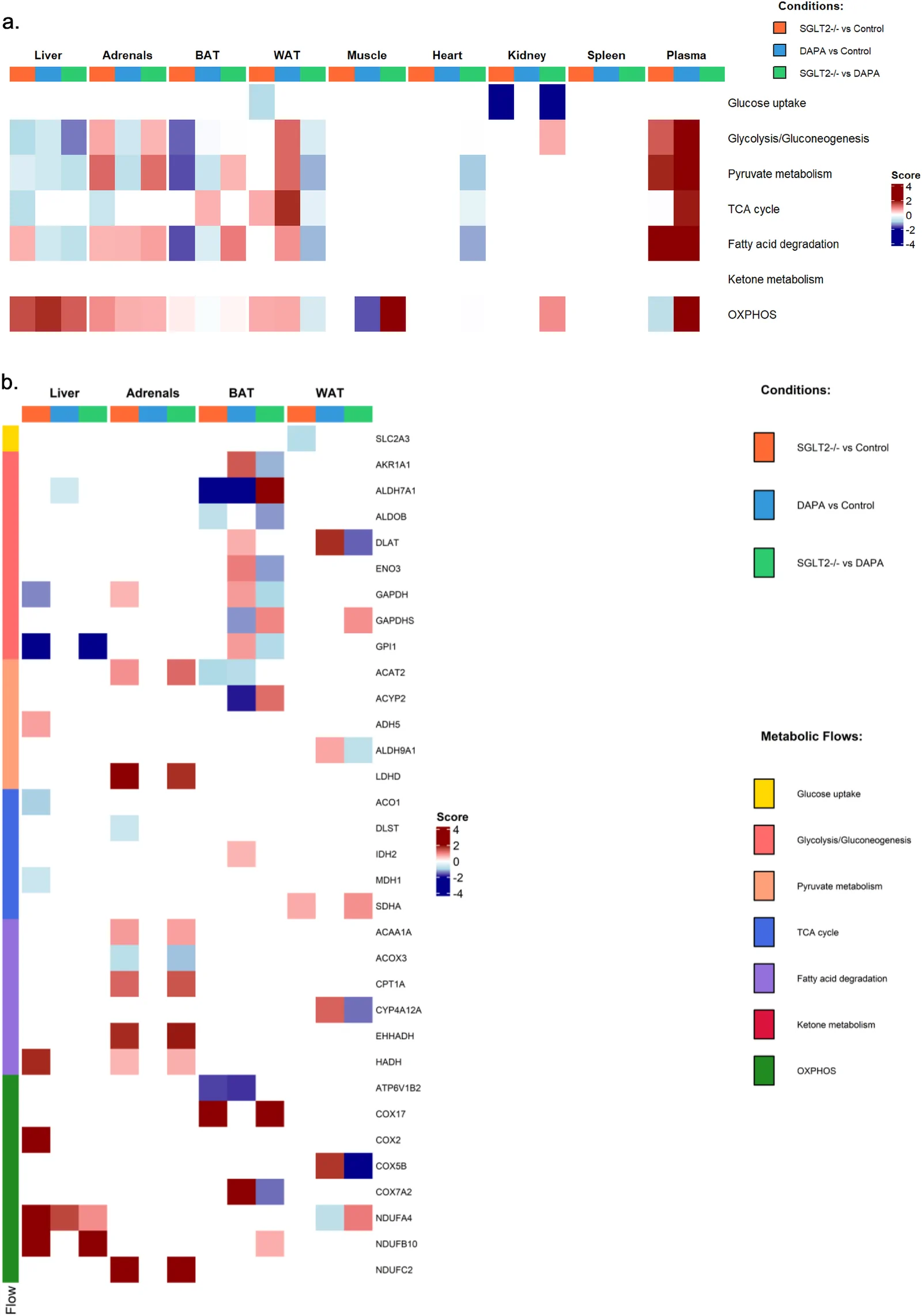
Metabolic pathway remodelling across the three comparisons (a) Pathway-level heatmap showing mean log_2_-fold changes of differentially expressed proteins (|log_2_FC| ≥ 0.5, adj. P < 0.05) across seven metabolic pathways (glucose uptake, glycolysis/gluconeogenesis, pyruvate metabolism, TCA cycle, fatty acid degradation, ketone metabolism, OXPHOS) in eight tissues and plasma. Conditions: SGLT2−/− vs control (orange), DAPA vs control (blue), SGLT2−/− vs DAPA (green). Blue indicates downregulation, red indicates upregulation (scale: −4 to +4). (b) Protein-level heatmap of the eight most enriched differentially expressed proteins per pathway, organised as contiguous metabolic blocks in the four tissues (Liver, Adrenals, BAT, WAT). Left annotation indicates metabolic pathway (colour-coded). Top annotation indicates condition. Same colour scale as panel (a).

### 2.11 Human dapagliflozin treatment identifies redox- and metabolic pathways overlapping with the preclinical findings

To validate our findings for cross-species translational value, we conducted an analysis of two proteomics data sets from human participants. Proteomic differential analysis was conducted on skeletal muscle biopsies and plasma samples obtained at week 24 from participants with type 2 diabetes randomised to the dapagliflozin (DAPA) or diet-control (DIET-CON) groups of the DETA trial [16]**.** The exercise intervention groups of the parent DETA trial were not included in the present proteomic analysis. Our analysis revealed a total of 114 DEPs in the muscle (Fig. 12a), of which 71 were upregulated (red), and 43 were downregulated (blue). Five key DEPs, *MCU, NDUFB10, TRIM63, DES*, and *MUSTN1,* are highlighted in Fig. 12a, which were validated at the transcript level using human primary isolated myocytes and RT-qPCR (Supplementary Fig. 1). The plasma revealed 41 DEPs, of which 22 were upregulated and 19 were downregulated (Fig. 12b). Enriched cluster ontological analysis was performed to identify the overarching functional theme of the dataset, revealing many oxidative stress-related terms in muscle, such as Krebs cycle disorders, aerobic respiration and respiratory electron transport, and in plasma, including biological oxidations and triglyceride catabolic processes (Fig. 12c and d). Longitudinal group × time analysis of skeletal-muscle protein abundance from baseline to week 24 identified significant enrichment of mitochondrial and energy-metabolism processes, including oxidative phosphorylation, electron transport and NADH dehydrogenase complex assembly (Fig. 12e). Cross-species comparison identified several overlapping redox- and mitochondrial-related GO biological processes between the human longitudinal response and dapagliflozin-treated mouse skeletal muscle (Fig. 12f). Importantly, this overlap indicates convergence at the pathway level and does not imply concordant direction of regulation between species. KEGG pathway analysis of the human muscle and plasma was provided mechanistic insights into the same neurodegenerative diseases and oxidative stress terms as highlighted in the mice proteomics (Supplementary Fig. 2). Baseline GSEA revealed pre-existing differences between the DAPA and DIET-CON groups, including negative enrichment of several mitochondrial and oxidative phosphorylation-related processes in the DAPA group (Supplementary Fig. 3). Accordingly, the longitudinal group × time findings should be interpreted in the context of this baseline imbalance.

**Fig. 12:**
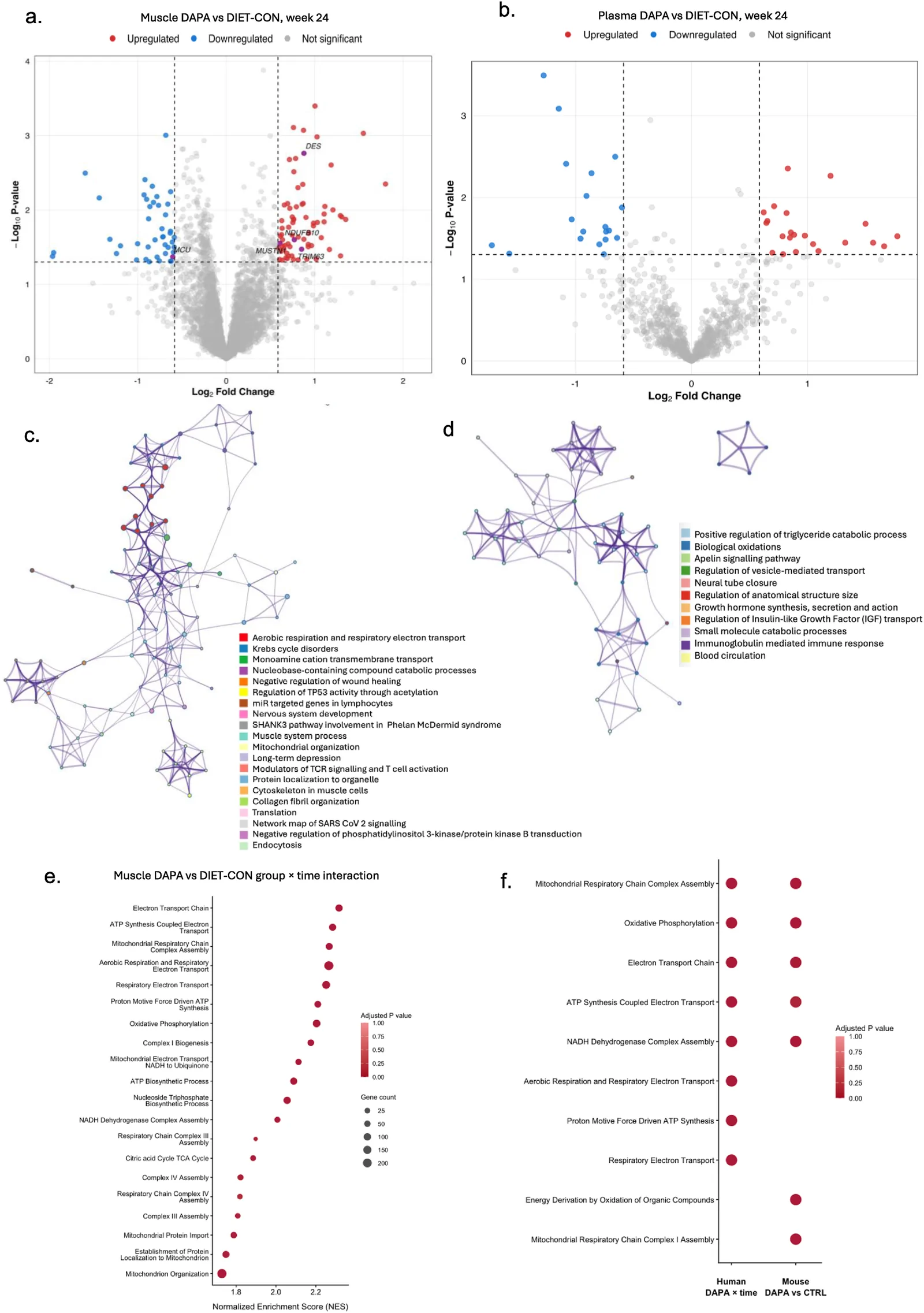
Comparative proteomic analysis of skeletal muscle and blood plasma from dapagliflozin-treated and diet-control participants with type 2 diabetes from the DETA trial. A volcano plot denoting significant DEPs in human (a) muscle and (b) plasma following differential analysis of dapagliflozin-treated patients (DAPA) and participants in the DIET-CON group after 24 weeks. Significantly upregulated DEPs are highlighted in red and those downregulated are highlighted in blue, with significance thresholds defined as p < -log_10_ (0.05) and log_2_ fold change > (1.5). Enrichment clusters of DEPs were created using Metascape (https://metascape.org) using GO and KEGG ontological pathways for human (c) muscle and (d) plasma using the post-24 week analysis, where individual nodes represent proteins that share the same ID cluster. (e) Gene Ontology enrichment analysis of significant pathways identified in the human muscle DAPA and DIET-CON group × time interaction analysis. Dot size indicates protein count and colour represents the adjusted p value. (f) Enrichment of oxidative stress-specific GO biological processes of human DAPA and DIET-CON group × time interaction and mouse DAPA vs CTRL muscle DEPs, ordered and coloured by significance of the adjusted p values. Pathway overlap does not necessarily indicate concordant direction of regulation between species.

To further investigate the oxidative stress-related pathways identified by proteomic analysis, we performed complementary molecular and biochemical analyses in selected tissues. Expression of the oxidative stress- and antioxidant-response genes Gpx1, Sod2, Hmox1, Gss and Prdx6 was assessed by RT-qPCR in liver, adrenal gland, WAT and BAT (Supplementary Fig. 4), revealing tissue- and intervention-specific differences in several targets. Liver 4-HNE, a marker of lipid peroxidation, was additionally assessed by Western blot but did not differ significantly between groups (Supplementary Fig. 5). Total liver glutathione was reduced in dapagliflozin-treated mice and was significantly reduced in SGLT2-null mice relative to controls (Supplementary Fig. 6). Finally, cytosolic and mitochondrial aconitase activities were assessed in BAT as redox-sensitive metabolic readouts. Cytosolic aconitase activity was significantly increased in SGLT2-null mice, whereas mitochondrial aconitase activity did not differ significantly between groups (Supplementary Fig. 7). Together, these analyses provide complementary evidence of altered redox-associated molecular and metabolic processes following genetic or pharmacological SGLT2 suppression, although they do not constitute direct measurements of in vivo ROS production.

## 3 Discussion

This study provides the first comprehensive multi-tissue proteomic characterisation of genetic and pharmacological inhibition of sodium–glucose cotransporter 2 (SGLT2), revealing a coordinated yet organ-specific metabolic remodelling across key metabolic tissues. While a previous multi-omics study in SGLT2-deficient models focused primarily on off-target effects [17], our data demonstrate that SGLT2 deletion itself induces a structured systemic response. The liver, adrenal glands, and brown adipose tissue (BAT) collectively account for the majority of tissue-specific proteomic changes, with plasma alone contributing a comparable number of differentially expressed proteins (DEPs). Functional enrichment consistently identified core metabolic pathways, including fatty acid, amino acid, and carbon metabolism, alongside oxidative stress-related processes such as oxidative phosphorylation and glutathione metabolism.

These tissue-specific responses likely reflect distinct physiological adaptations to chronic SGLT2 loss. The liver exhibited a coordinated shift toward oxidative metabolism, characterised by upregulation of mitochondrial electron transport chain components, peroxisomal and xenobiotic detoxification pathways, alongside downregulation of glycolytic enzymes, aligning with the “thrifty substrate” hypothesis of SGLT2 inhibition [18]. The adrenal glands showed a similar metabolic profile, with additional upregulation of steroidogenesis, fatty acid metabolism and glutathione pathways, suggesting substantial adrenal metabolic and steroidogenic remodelling following SGLT2 deletion. This is consistent with our previous phenotypic characterisation of these animals, in which SGLT2 deletion restored a more physiological pattern of corticosterone secretion, with a less pronounced effect observed following dapagliflozin treatment [15]. In complementary experiments using the human adrenocortical H295R cell line, we previously demonstrated that SLC5A2 expression is responsive to inflammatory and anti-inflammatory cytokine signalling and that pro-inflammatory cytokines can modulate expression of key steroidogenic enzymes [15]. Together, these observations support a relationship between SGLT2-associated signalling, inflammatory regulation and adrenal steroidogenesis. However, the bulk-tissue proteomic approach used in the present study cannot resolve the cellular localisation of SGLT2 within the adrenal gland, and defining its adrenal cell-type-specific expression and function will require dedicated spatial or cell-specific studies.

In contrast, BAT displayed widespread downregulation of mitochondrial and metabolic pathways, including ketone and branched-chain amino acid metabolism, as well as cytoskeletal processes, indicating impaired thermogenic capacity rather than adaptive remodelling. This aligns with previous evidence that glucose-derived tricarboxylic acid (TCA) cycle intermediates are essential for thermogenesis [19].

Although a previous study reported similar increases in ketone utilisation and altered substrate metabolism in SGLT2-deficient and inhibitor-treated models, genetic deletion did not fully recapitulate the cardioprotective effects observed with pharmacological SGLT2 inhibition [7]. The same study observed the accumulation of acylcarnitines, broadly linked to impaired fatty acid oxidation, and dysregulation of mitochondrial fuel utilisation, both of which have been reported in patients with heart failure and other cardiac disorders [20], [21], [22]**.** Our heart proteomics revealed downregulation of fatty acid oxidation, ketone body metabolism, branched-chain amino acid degradation, and redox-associated pathways, implying impaired rather than adaptive cardiac metabolic remodelling under SGLT2 deletion.

Plasma proteomics further highlighted the systemic nature of these adaptations, with enrichment of fatty acid, amino acid, and xenobiotic metabolism pathways, alongside one-carbon metabolism and PPAR signalling. The upregulation of liver-specific enzymes such as MAT1A suggests enhanced S-adenosylmethionine (SAM) synthesis, consistent with previous reports linking SGLT2 loss to altered methyl metabolism and suppression of inflammatory signalling via NF-κB [10]**.** This is further supported by reductions in pro-inflammatory gene expression observed in related models [10], [15], aligning with our preliminary findings in plasma and adipose tissue.

A central finding of this study is the consistent enrichment of oxidative stress-related pathways across both tissue and plasma in SGLT2-null and dapagliflozin-treated models. A substantial proportion of shared DEPs were associated with the regulation of reactive oxygen species (ROS), mitochondrial respiration, and glutathione metabolism. These proteomic findings were further examined using complementary molecular and biochemical measures of redox regulation. Expression of oxidative stress- and antioxidant-response genes, including Gpx1, Sod2, Hmox1, Gss and Prdx6, was altered across liver, adrenal gland, WAT and BAT, supporting tissue-specific modulation of antioxidant and redox-associated processes. Total liver glutathione was reduced, reaching significance in SGLT2-null mice, whereas liver 4-HNE abundance was not significantly altered, indicating no detectable change in this marker of lipid peroxidation. In BAT, cytosolic aconitase activity was significantly increased in SGLT2-null mice, whereas mitochondrial aconitase activity was not significantly altered. Given the sensitivity of aconitase iron-sulfur clusters to reactive species, these measurements provide an additional redox-sensitive metabolic readout, although they do not directly quantify ROS production [23]. Collectively, these findings support perturbation of redox-associated pathways following both genetic and pharmacological SGLT2 suppression but suggest that these responses are tissue- and pathway-specific rather than reflecting a uniform increase or decrease in oxidative stress. As ROS were not captured at the time of tissue collection, the present experiments cannot establish changes in in vivo ROS production. Future studies incorporating direct or appropriately stabilised measurements of ROS will therefore be required to determine whether the observed molecular changes reflect altered ROS generation, antioxidant adaptation, or both.

A key observation is that a substantial proportion of DEPs identified in dapagliflozin-treated mice were associated with oxidative stress-related processes, further supporting redox regulation as a prominent component of the molecular response to dapagliflozin. Dapagliflozin has been shown to enhance β-oxidation and ketone metabolism across multiple tissues, partly via activation of SIRT1 and improved mitochondrial biogenesis [24], [25]. In the liver, these changes are consistent with attenuation of Metabolic Dysfunctional-Associated Steatotic Liver Disease (MASLD) through PPARα-mediated pathways [26]. The broader, predominantly downregulated proteomic profile observed with dapagliflozin, compared with the more localised effects of SGLT2 deletion, may reflect both systemic metabolic adaptation and additional pharmacological effects beyond SGLT2 inhibition. Importantly, the genetic and pharmacological models should be considered complementary rather than equivalent approaches to SGLT2 suppression. Previous phenotypic characterisation of these animals demonstrated comparable glycosuria between SGLT2-null and dapagliflozin-treated mice, but greater improvements in glucose tolerance and insulin sensitivity following genetic deletion, together with more pronounced effects on corticosterone regulation [15]. Lifelong genetic SGLT2 deletion may induce chronic or developmental adaptations that differ from those produced by short-term pharmacological inhibition. Consequently, differences between the SGLT2-null and dapagliflozin-treated groups cannot be attributed exclusively to SGLT2-independent actions of dapagliflozin.

The comparison between SGLT2-null and dapagliflozin-treated mice revealed more DEPs than either intervention alone, relative to vehicle-treated controls, indicating substantial divergence between the proteomic responses to genetic SGLT2 loss and pharmacological inhibition. The most pronounced differences occurred in the kidney and heart, tissues that showed comparatively limited changes in the individual comparisons with the control. The emergence of the kidney is particularly notable given that it is the primary site of SGLT2 expression; indeed, SGLT2 was identified as a DEP exclusively in the kidney across our proteomic dataset, consistent with its established tissue distribution. Kidney enrichment among oxidative stress-related terms was restricted predominantly to mitochondrial respiratory processes, with a higher abundance of associated proteins in SGLT2-null than in dapagliflozin-treated mice. This is consistent with previous reports of reduced oxidative phosphorylation-associated transcriptional programmes in proximal tubular epithelial cells following dapagliflozin treatment [27]. These findings suggest divergent renal metabolic responses to genetic SGLT2 loss and pharmacological inhibition, with differences involving glycolysis/gluconeogenesis, oxidative phosphorylation and glucose-handling pathways.

These findings should be considered in the context of the canonical function of SGLT2 in renal glucose reabsorption. Although SGLT2-dependent glucose transport was not measured directly, the kidney-specific differential abundance of SGLT2/SLC5A2 and the altered renal glucose-uptake-associated module are consistent with the kidney as the principal site of its direct glucose-transport effect. In contrast, no significant differential abundance of SGLT1/SLC5A1, GLUT1/SLC2A1 or GLUT4/SLC2A4 was detected across the tissue proteomic datasets. Together with the extensive metabolic remodelling observed in extra-renal tissues, these findings suggest that the multi-organ proteomic responses cannot be explained solely by changes in local SGLT2-dependent glucose entry and may also reflect downstream systemic metabolic adaptations and/or additional pharmacological effects of dapagliflozin.

Whether these differences reflect chronic adaptation to lifelong SGLT2 loss, effects of short-term dapagliflozin treatment on renal substrate metabolism, or a combination of both cannot be determined from the present study. Divergent responses were also evident in other tissues. In skeletal muscle, dapagliflozin was associated with suppression of oxidative phosphorylation that was not reproduced by genetic deletion. At the same time, the heart exhibited reduced pyruvate metabolism, TCA-cycle and fatty-acid degradation specifically in the direct comparison, highlighting cardiac metabolic responses that distinguish pharmacological inhibition from genetic SGLT2 loss.

Recent evidence further supports SGLT2-independent metabolic actions of SGLT2 inhibitors in the heart. Chen et al. compared empagliflozin treatment in wild-type and SGLT2-deficient mice with heart failure and demonstrated cardioprotection in both genotypes, accompanied by changes in glycolysis, SIRT3/ERRα signalling, ATP production and mitochondrial metabolic pathways, including the TCA cycle, fatty acid and branched-chain amino acid metabolism, and respiratory chain complexes [28]. These findings are consistent with our observation that pharmacological SGLT2 inhibition elicits molecular responses that are not fully recapitulated by genetic SGLT2 deletion. Importantly, our multi-organ analysis extends this concept beyond the heart, revealing tissue-specific differences between genetic and pharmacological SGLT2 suppression across kidney, skeletal muscle, adipose tissue, adrenal glands and other metabolically relevant tissues.

Importantly, cross-species comparison with the human DETA datasets identified convergence at the pathway level, particularly in processes related to mitochondrial respiration and cellular energetics. In skeletal muscle, longitudinal analysis of the DAPA and DIET-CON groups identified significant enrichment of oxidative phosphorylation, respiratory electron transport, NADH dehydrogenase complex assembly and ATP-related processes. Several of these pathways also emerged from the murine skeletal-muscle proteome, supporting mitochondrial energy metabolism as a shared biological domain responsive to dapagliflozin across species. However, pathway overlap did not necessarily correspond to concordant direction of regulation: oxidative phosphorylation-related proteins were reduced in dapagliflozin-treated mouse skeletal muscle relative to control, whereas the human group × time analysis indicated relative enrichment over 24 weeks in DAPA compared with DIET-CON. These findings therefore support cross-species convergence on mitochondrial and redox-associated processes rather than direct replication of the molecular response. Differences in species, metabolic context and treatment duration, together with the baseline imbalance in mitochondrial pathways in the human cohort, may contribute to the divergent directional responses. The human findings should consequently be considered supportive translational evidence that the pathways identified in the murine analysis are relevant to dapagliflozin treatment in humans, rather than evidence of an identical cross-species response.

This study has several limitations. First, the murine models reflect chronic genetic deletion or controlled pharmacological inhibition, which may not fully capture the temporal dynamics and clinical heterogeneity of SGLT2 inhibitor use in humans. Second, proteomic analyses provide associative insights and do not establish causality between SGLT2 modulation, oxidative stress, and metabolic outcomes. Third, murine tissue sampling was limited to male mice. Additionally, the human proteomic findings should be interpreted as supportive cross-species evidence, as the relatively small DETA proteomic subset was not individually matched for demographic and clinical characteristics and included an imbalance in sex distribution between treatment groups, as well as a difference in fat-free mass. Baseline differences were also evident in mitochondrial and oxidative phosphorylation-related pathways in skeletal muscle, which should be considered when interpreting the longitudinal pathway responses despite use of the group × time interaction. Finally, potential off-target effects of dapagliflozin cannot be fully excluded and may contribute to the broader proteomic changes observed compared to genetic deletion models. Future studies will aim to define the causal role of oxidative stress modulation in mediating the systemic effects of SGLT2 inhibition in males and females. Longitudinal and time-resolved analyses will be critical to distinguish early adaptive responses from sustained metabolic reprogramming. Finally, targeted manipulation of redox pathways may help determine whether modulation of oxidative stress can replicate or enhance the therapeutic benefits of SGLT2 inhibitors in multi-organ disease.

## 4 Conclusion

In summary, this study demonstrates that SGLT2 inhibition -whether genetic or pharmacological-drives a coordinated, multi-organ metabolic reprogramming characterised by a conserved oxidative stress and mitochondrial signature. By integrating comprehensive proteomic profiling of tissues and plasma with cross-species comparison, we identify redox regulation and mitochondrial metabolism as prominent, recurrent biological processes associated with SGLT2 modulation. The liver and adrenal glands emerge as key metabolic hubs orchestrating this response, while peripheral tissues exhibit distinct adaptive adjustments, collectively contributing to whole-body metabolic homeostasis. The overlap of these pathways with human datasets strengthens the translational relevance of the findings while highlighting mitochondrial and redox-associated processes as candidate mechanisms for further investigation. These insights position oxidative stress modulation as a potential therapeutic target and provide a mechanistic framework for understanding -and potentially expanding-the use of SGLT2 inhibitors in multi-organ disease.

## 5 Methods

### 5.1 Animals

Tissue and plasma samples analysed in this study were derived from mice previously phenotypically characterised in Brisnovali et al. [15]. The multi-organ tissue proteomic profiling and comparative analyses presented here were performed for the present investigation and have not previously been reported; plasma proteomics from these animals was previously reported and was re-analysed here using the analytical framework applied to the tissue datasets.

All animal procedures were approved by the British Home Office under the UK Animal (Scientific Procedures) Act 1986, with approval from the local ethical committee (Animal Welfare and Ethics Review Board, AWERB), at the Central Biological Services (CBS) unit at the Hammersmith Campus of Imperial College London. Adult male C57BL/6J mice (Envigo, Huntingdon, UK) were maintained under controlled temperature (21-23°C) and light (12:12 h light-dark schedule, lights on at 0700). As this study utilised existing biopsies, male mice were used.

SGLT2-null mice were generated on a C57BL/6J genetic background by CRISPR/Cas9-mediated deletion of exon 1 of Slc5a2, as previously described [11]. Heterozygous animals were intercrossed to generate wild-type, heterozygous and homozygous littermates [11].

From the age of 8 weeks, C57BL/6J control and SGLT2 null mice were placed on a 58 kcal% Fat and 18.4% Sucrose diet (D12331, Research Diet, New Brunswick, NJ) to induce obesity and diabetes. Sample size calculation was performed with blood glucose as a primary outcome. Randomisation was performed according to pre-treatment body weight. Group 1 (n = 5) received 10 mg/kg dapagliflozin dissolved in methylcellulose daily for 14 days, through oral gavage at 0800. The metabolic phenotypes of these animals have been characterised previously [15]. Both SGLT2-null and dapagliflozin-treated mice exhibited marked glycosuria, with no significant difference in urinary glucose/creatinine ratio between the two interventions, and both restored the glucose-stimulated GLP-1 response. However, the models were not metabolically equivalent: SGLT2-null mice showed greater improvements in glucose tolerance and insulin sensitivity than dapagliflozin-treated mice despite similar glycosuria, while neither intervention produced significant weight loss. SGLT2-null mice also exhibited more pronounced changes in corticosterone regulation and adipose Hsd11b1 expression [15]. Comprehensive cardiac and renal functional phenotyping was not performed in these animals.

Although mice underwent fasting for metabolic phenotyping procedures as previously described [8], the tissue and plasma samples used for the present proteomic analyses were subsequently collected in the fed state and immediately snap-frozen at −80°C.

### 5.2 DAPA randomised clinical trial

Human plasma and skeletal muscle samples were obtained from participants enrolled in the Dapagliflozin, Exercise Training and physicAl function (DETA) randomised controlled trial [16]. As described in the published DETA protocol, an optional skeletal muscle biopsy substudy collected fasted vastus lateralis biopsies at baseline and at week 24. For the present study, proteomic analyses were performed on time-matched skeletal muscle biopsy and EDTA-plasma samples from participants in the Diet Control (DIET-CON) and dapagliflozin (10 mg once daily; DAPA) groups at week 24. Participant characteristics for the subset included in the proteomic analyses are presented in Supplementary Table 3. These samples were derived from randomised treatment groups within the DETA trial and were not individually matched for demographic or clinical characteristics. Owing to the relatively small number of participants available for the present proteomic analysis, an imbalance in sex distribution was present between groups, and fat-free mass also differed between groups.

### 5.3 Primary skeletal muscle myoblasts

Primary skeletal muscle myoblasts were isolated from skeletal muscle biopsies (n = 5) from healthy individuals, as previously described [29]**.** At passage 1, HDMC cultures were subjected to immunocytochemistry analysis for the calculation of Desmin positivity to provide an insight into the isolated cellular population. Combined Desmin positivity across all isolated populations was calculated at 68.2% ± 5.4 at passage 1. Myoblasts were proliferated to sub-confluence in growth medium (GM) (Ham's F-10 Nutrient Mix supplemented with 1% Penicillin-Streptomycin (PS) and 20% Foetal Bovine Serum) at which point they were passaged and re-plated on collagen-coated 6-well plates (at 37°C under humidified 95% air and 5% CO2) at 50,000 cells/well and proliferated for 3-5 days. Upon reaching confluency, differentiation into myotubes was induced using differentiation media (DM) (Dulbecco's Modified Eagle Medium supplemented with 1% Penicillin-Streptomycin and 2% Horse serum) and cultured for a maximum of 10-14 days. Myotubes were treated with dapagliflozin at concentrations of 10 μM or 10 nM (diluted in DM), alongside an untreated control, for 24 h. Following dosing, cells were harvested using 500 μL TRIzol per well and stored in separate aliquots at −80°C.

### 5.4 RNA extraction, cDNA synthesis and qPCR

RNA was extracted from skeletal muscle myoblasts by resuspending them in TRIzol reagent (Ambion, Life Technologies, USA) and chloroform (Sigma-Aldrich, USA). Total RNA isolation was performed using the TRIzol Plus RNA Purification Kit (Applied Biosystems, ThermoFisher, USA) according to the manufacturer's protocol. The High-Capacity cDNA Reverse Transcription kit (Applied Biosystems, ThermoFisher, USA) was used for cDNA synthesis. Master mix for quantitative PCR (qPCR) was made using SYBR Green (Applied Biosystems, ThermoFisher, USA). RNase samples were analysed using the 7500 Fast Real-Time PCR system (Applied Biosystems, serial no. 275010654). All data were normalised against UBC as the housekeeping gene, and all ΔCt values were calculated using the average from dual replicates. The 2^−ΔΔCt^ analytical method was used. The primers used are shown in Supplementary Table 2.

*Western blot: T*issue samples were digested in RIPA buffer, supplemented with Halt Protease & Phosphatase Inhibitor Cocktail [1:100] (ThermoFisher, 78440). Protein concentrations were measured using a BCA assay, in which 30 μg of protein was supplemented with 1/4 volume of Laemmli buffer and boiled at 95°C for 5 min. Samples were then run on a 12.5% SDS gel at 120V for 90 min, following a blot transfer to PVDF (Bio-Rad) at 90V for 1 h on ice. The blots were blocked in 5% milk in TBST for 1 h at room temperature, followed by 3 washes in TBST for 10 min and an overnight incubation with the primary 4-HNE anti-mouse (ThermoFisher, BLR303 M) antibody in 5% milk in TBST overnight at 4°C. The next day, blots were again washed in TBST and incubated with the secondary anti-mouse rabbit antibody (Sigma, **source**) for 1 h at room temperature. Following a brief rinse in TBST, the blot was coated with ECL detection buffer (Thermo Scientific, SuperSignal West Pico PLUS Chemiluminescent Substrate, 34577) at a 1:1 ratio before being imaged on the ChemiDoc scanner.

*Glutathione assay:* Using the protocol of the GSH Assay Kit (Abcam, ab239709), 100 mg of liver tissue was prepared in 0.4 mL Glutathione Reaction Buffer + 100 μL 5% SSA. GSH standards were prepared alongside samples. 160 μL of the reaction mix containing NADPH Generating Mix, Glutathione Reductase, and Glutathione Buffer was loaded into a 96-well plate and incubated for 10 min to generate NADPH. 20 μL of samples and standards were loaded into the corresponding wells and incubated for 10 min 20 μL of substrate solution was added and incubated for 5-10 min, before measuring the absorbance at OD 405 nm. GSH concentrations in the sample were calculated using the glutathione calibration curve.

*Aconitase assay:* Using the protocol of the Aconitase Assay Kit (Abcam, ab83459), 40 mg of BAT was homogenised in 100 μL Assay Buffer 7 and centrifuged at 4°C at 800 xg for 10 min to remove insoluble material, and then again at 17,000 xg at 4°C for 15 min to separate the c-aconitase (supernatant) and the m-aconitase, where the latter was a pellet resuspended in 100 μL Assay Buffer 7. Samples were activated with 10 μL of a mixture of equal parts Cysteine Standard and Ferrous Ammonium Sulfate for 1 h on ice. Isocitrate Standards were prepared alongside the samples, and 50 μL was added to each corresponding well in a 96-well plate. A 50 μL reaction mix containing 42 μL Assay Buffer 7, 6 μL Enzyme Mix VII and 2 μL Substrate XI was added to each well and incubated at room temperature for 1 h 10 μL of Developer Solution II was added to each well and incubated at room temperature for 10 min before measuring the absorbance at OD 450 nm on a microplate reader. Aconitase activity was calculated according to the protocol booklet and expressed as nmol/min/mL.

### 5.5 Mouse plasma proteomics analysis

Proteomics analysis was conducted by Seer Inc. (California, USA) through Liquid-Chromatography-Mass Spectrometry (LC-MS).

*Sample preparation for direct digest protocol:* For direct digestion of neat plasma samples, proteins were denatured, reduced, alkylated and subjected to proteolytic digestion (Trypsin and Lys-C) for 3 h at 37°C. Peptides were purified by solid phase extraction, and yields were determined (Applied Biosystems, ThermoFisher).

Sample preparation with the Proteograph™ XT Assay Protocol: Samples were processed with the Proteograph XT Assay [30]. In brief, plasma proteins were quantitatively captured in nanoparticle (NP) associated protein coronas. Proteins were subsequently denatured, reduced, alkylated, and subjected to proteolytic digestion (Trypsin and LysC). Peptides were purified, and yields were determined. Peptides were dried down overnight using a vacuum concentrator and reconstituted with a reconstitution buffer to a concentration of 50 ng/μL.

*Data-Independent Acquisition LC-MS/MS:* For Data-Independent Acquisition (DIA), 8 μL of reconstituted peptide mixture from each NP preparation was analysed, resulting in a constant 400 ng mass MS injection between NP A and NP B samples. Each sample was analysed with a Vanquish NEO nanoLC system coupled with an Orbitrap TM Astral TM (Applied Biosystems, ThermoFisher) mass spectrometer using a trap-and-elute configuration. First, the peptides were loaded onto an AcclaimTM PepMapTM 100 C18 (0.3 mm ID x 5 mm) trap column and then separated on a 50 cm μPACTM analytical column (PharmaFluidics) at a flow rate of 1 μL/min using a gradient of 5–25% solvent B (0.1% FA, 100% ACN) mixed into solvent A (0.1% FA, 100% water) over 22 min, resulting in a 30-min total run time. The mass spectrometer was operated in DIA mode with MS1 scanning and MS2 precursor isolation windows between 380 and 980 *m*/*z*. MS1 scans were performed on the Orbitrap detector at 240,000 R every 0.6 s, with a 5 ms ion injection time or a 500% AGC (500,000 ion) target. Two-hundred fixed window MS2 DIA scans were collected at the Astral detector per cycle with 3Th precursor isolation windows, 25% normalised collision energy, and 5 ms ion injection times with a 500% (50,000 ion) active gain control maximum. MS2 scans were collected from 150 to 2,000 m/z.

### 5.6 Mouse tissue proteomics analysis

Proteomics analysis was conducted by the Leicester van Geest Multi-OMICS Facility, through Liquid-Chromatography-Mass Spectrometry (LC-MS).

*Sample preparation:* The tissue was homogenised with silica beads in ammonium bicarbonate, pH 7.6, with 0.5% ammonium deoxycholate and then centrifuged at 14,000 xg to remove residual tissue debris. The supernatant was removed, and proteins were then reduced using dithiothreitol (DTT), alkylated with iodoacetamide (IAA) and subjected to proteolytic digestion at a 1:25 trypsin: protein ratio overnight at 37°C. Peptides were then desalted using solid phase extraction, and their relative concentrations determined by the *o*-phthalaldehyde (OPA) assay. The peptides were diluted to 500 ng/μL and spiked with 100 fmol/μL alcohol dehydrogenase (ADH) as a standard. 2 μL of sample was analysed using the Waters PLC Synapt G2S MS, corresponding to 1 μg of sample with 100 fmol ADH.

*Data-Independent Acquisition LC-MS/MS:* Proteomic analysis was performed using Progenesis QI for Proteomics version 4.2 (Nonlinear Dynamics, Waters Corporation, UK). Spectral data were searched against the UniProt *Mus musculus* FASTA database for peptide and protein identification. Trypsin was designated as the proteolytic enzyme and up to two missed cleavage sites were allowed. Carbamidomethylation of cysteine was added as a fixed modification. Identification thresholds were set to at least three fragment ions per peptide, seven fragment ions per protein, and a minimum of two peptides per protein. The false discovery rate (FDR) was rigorously maintained below 1% for both peptide and protein identifications. Protein quantification was based on the Hi3 approach utilising the three most intense peptides per protein. The finalised data outputs were then transferred to Microsoft Excel for downstream statistical evaluations and bioinformatics interpretation.

### 5.7 Human plasma and muscle proteomics analysis (DAPA RCT)

DAPA RCT proteomic analysis was conducted by the Leicester van Geest Multi-OMICS Facility using the timsTOF HT mass spectrometer coupled to the Evosep ONE LC.

*Plasma sample preparation:* Plasma was prepared as described by Maxwell et al. [31]*.* Plasma samples corresponding to 100 μg protein were adjusted to pH 7.8 using 50 mM ammonium bicarbonate (AmBic) and were processed using an automated AssayMAP in-solution digestion workflow. Samples were denatured with rapiGest (Waters, Milford, CT, USA) and reduced using dithiothreitol (DTT) at 65°C for 30 min. This was followed by an alkylation using iodoacetamide (IAA) for 30 min. Following dilution, samples were digested with Trypsin/Lys-C (Promega, Southampton, UK) at a 1:25 ratio at 37°C for 16 h. Peptides were acidified using 1% formic acid (FA), cleaned using C18 cartridges (Agilent Technologies, Santa Clara, CA, USA) and eluted in 90% and subsequently 70% acetonitrile (MeCN) with 50 μL 0.1% FA. Samples were dried in vacuo and stored at – 80°C until required.

*Muscle sample preparation:* Muscle samples were lysed in 500 μL RIPA lysis buffer (25 mM Tris-HCl, pH 7.6, 150 mM NaCl, 1% NP-40, 1% sodium deoxycholate, 0.1% SDS, Thermo Scientific, Loughborough, UK) with a protease inhibitor cocktail (PIC, Roche, Welwyn, UK) and homogenised using steel beads (Benchmark Scientific, New Jersey, USA). A volume of lysate corresponding to 10 μg protein per sample was determined by bicinchoninic acid (BCA) assay, followed by normalisation to pH 7.8 in AmBic. Samples underwent reduction, alkylation, digestion, and clean-up for bottom-up proteomics following the single-pot, solid-phase-enhanced (SP3) sample preparation [32], [33]. Reduction and alkylation were performed simultaneously using 10 mM Tris(2-carboxyethyl)phosphine (TCEP), 40 mM chloroacetamide (CAA) at 95°C for 5 min. Proteins were bound to Mag SpeedBeads Carboxylate Magnetic Beads (Cytiva Life Sciences, Buckinghamshire, UK) and washed using acetonitrile and 80% ethanol. Bound peptides were subjected to enzymatic digestion using trypsin gold/lysC mix (Promega, Southampton, UK) at a concentration of 1:25 at 37°C for 16 h. Peptides were acidified with trifluoroacetic acid (TFA) to a final concentration of 1% acid and eluted from the magnetic beads.

*Evotip Loading:* Peptide concentrations were quantified via *o*-phthalaldehyde (OPA) assay (Pierce Quantitative Fluorometric Peptide Assay; Thermo Scientific, Loughborough, UK) and normalised to 500 ng. 20 μL of the samples were automatically loaded onto Evotips using an advanced Agilent Bravo AssayMAP, performed as previously described with Evotip-specific hardware. Samples were subsequently activated with propanol and equilibrated using solvent A, followed by automated sample loading via VWorks [31].

*LC-MS/MS Analysis:* Peptides were analysed using an Evosep ONE LC coupled to a timsTOF HT mass spectrometer. Separation was performed on a C18 column with a 22.5 min gradient (0.1% FA/MeCN + 0.1% FA). Data were acquired using DIA-PASEF with optimised windows (392–1017 *m*/*z*, 0.67–1.3 Vs cm^−2^), 15 scans per cycle (∼1.06 s), and processed in real time using ProteoScape [31].

*Data Processing:* Raw data were processed using DIA-PASEF Spectronaut version 20.5. Database searching was performed against the *homo sapiens* reviewed FASTA using directDIA mode with BGS factory settings using enzyme cleavage rules for Trypsin/P, LysC.

### 5.8 Statistical analyses

*Data pre-processing and differential expression analysis:* Principal component analysis (PCA) was performed to assess initial separation among the three experimental conditions (Ctrl, Dapa and SGLT2^−/−^) using R Studio version 4.2.2. The quality control (QC) samples were initially excluded from the data pre-processing stages. Near-zero variance (NVZ) was removed using Caret, which removes samples with >90% identical values to mitigate multiple testing burdens. Zero values were imputed with the half minimum value within the same experimental condition to handle missing values for the following Log2 transformation to assume a Gaussian distribution. Following this, the unsupervised spectral feature selection function (SPECU, in Rdimtools) was implemented to use the 4th quartile as a negative control for RUVIII, alongside the QC samples as a reference, since these proteins are assumed to be the least variable and biologically relevant. RUVIII (in package ruv) was then used to remove unwanted variation to create a QC replicate corrected expression matrix. ARSyNSeq was used as a data cleaning tool to remove batch effects and unmeasured systematic noise picked up by G2S MS. The data was then normalised using TMM and transformed using Voom to adjust the analysis for the size of the input library and estimate the mean-variance of each gene based on its expression and stabilise variance, respectively. Using the linear models for microarray (limma) package, differential expression of proteins across experimental conditions (Ctrl, Dapa and SGLT2^−/−^) was calculated and represented as a log2 fold change, with a significance threshold of -log10(Benjamini-Hochberg adjusted p value) of 0.05. RLE plots were generated to highlight unwanted variation, whilst heatmaps were made to visualise the distribution of protein expression in each mouse and across the various conditions.

The differential expression analysis for murine plasma omitted the SPECU and RUViii cleaning stages of the analysis due to the lack of a QC control, as the plasma proteomics was conducted independently by Seer Inc.

*Pathway enrichment analysis:* Differentially expressed proteins (DEPs) identified by the differential limma analysis were converted to MGI symbols, using the analysis script available at https://doi.org/10.5281/zenodo.19000356, and organised by tissue and comparison into an Excel file. Plasma was analysed separately. All pathway enrichment and statistical analyses were performed using R (v4.4.2).

To assess potential pre-existing differences between the human treatment groups, baseline (week 0) skeletal-muscle proteomic data were additionally analysed using limma. A linear model was fitted to the baseline samples and differential protein abundance between the DAPA and DIET-CON groups was assessed using a specified contrast followed by empirical Bayes moderation. The resulting baseline differential-protein analysis was subsequently used for pathway enrichment analysis (Supplementary Fig. 3).

Tissue-level DEPs overlap was assessed using UpSet plots (UpSetR). KEGG and GO Biological Process over-representation analysis were performed using clusterProfiler (enrichKEGG and enrichGO, respectively) with org.Mm.eg.db as the annotation database for mouse (*Mus musculus*) and org.Hs.eg.db as the annotation database for humans (*Homo sapiens*), using the hypergeometric test with Benjamini–Hochberg (BH) correction (pvalueCutoff = 0.1, qvalueCutoff = 0.2) [34]. A minimum of 3 proteins per term was required, and terms with raw p-value < 0.01 were retained for visualisation, following Metascape thresholds [14]. Results were visualised as bar charts and/or tissue-resolved dot plots, with bar/bubble size proportional to protein count and colour intensity representing significance, –log10(adj. P-value) in the bar charts or direction ratio in dot plots. The direction ratio was calculated according to the logFC of all proteins enriched in that term, and the colour gradient ranges from blue = −1 (all downregulated) to red = 1 (all upregulated).

Pathway enrichment was performed using Metascape (www.metascape.org) with default parameters and networks being visualised in Cytoscape (v3.10). The Sankey diagrams were constructed from Metascape output (GO_AllLists.csv) to display the top 10 GO Biological Process terms with Benjamini–Hochberg correction ranked by total protein count. Line thickness was proportional to the percentage of proteins mapping to each term per tissue (%InGO), organ and pathway boxes were sized proportionally to total protein count, and pathway boxes were coloured by the organ contributing the most proteins. Diagrams were generated per comparison using ggplot2 and ggforce.

Pathway-level heatmaps were generated to visualise metabolic remodelling across tissues and conditions. Gene lists for seven metabolic pathways were defined: glucose uptake (custom gene set: SLC5A2, SLC2A1–SLC2A14), glycolysis/gluconeogenesis (KEGG mmu00010), pyruvate metabolism (KEGG mmu00620), TCA cycle (KEGG mmu00020), fatty acid degradation (KEGG mmu00071), ketone metabolism (KEGG mmu00072), and oxidative phosphorylation (KEGG mmu00190). Pathway scores were calculated as the mean log_2_-transformed fold change of all detected differentially expressed proteins within each pathway for a given tissue and condition. Scores were colour-mapped to distinguish between downregulated (blue) and upregulated (red) pathways. Heatmaps were stratified by tissue (liver, adrenals, BAT, WAT, muscle, heart, kidney, spleen, plasma) and arranged side-by-side to compare three conditions: SGLT2^−/−^ versus wild-type control (orange), dapagliflozin versus control (blue), and SGLT2^−/−^ versus dapagliflozin (green). Visualisation was performed using R (v4.x) with ComplexHeatmap (v2.x) and circlize packages.

Protein-level heatmaps were constructed to examine individual protein changes underlying metabolic pathway shifts. The eight most enriched differentially expressed proteins per pathway (ranked by mean absolute log_2_FC) were selected to reduce dimensionality while retaining interpretability. Left-hand annotation bars colour-coded each protein by its metabolic pathway; top annotation bars colour-coded columns by condition. The same diverging colour scale was applied, as previously mentioned. Heatmaps were generated in R using the ComplexHeatmap and tidyverse packages. Pathway gene sets were retrieved dynamically from the KEGG database via KEGGREST.

Cross-comparison overlap between conditions was illustrated by a Venn diagram (VennDiagram) and shared DEPs were annotated against a curated oxidative stress GO term list (Supplementary Table 1) via biomaRt and GO enrichment on common DEPs was performed using enrichGO from clusterProfiler.

Oxidative stress-related DEPs were defined by annotation to GO terms included in the Supplementary Table 1, including oxidative stress, ROS response, oxidative phosphorylation, antioxidant defence, mitochondrial function, ferroptosis, and DNA damage. The top 20 oxidative stress DEPs heatmap used the original limma results fold-change, displayed as a z-score heatmap with hierarchical clustering (pheatmap).

***Data and resource availability:*** The mass spectrometry proteomics data have been deposited in the ProteomeXchange Consortium via the PRIDE repository and will be made publicly available upon publication. Reviewer access credentials are provided in the Supplementary Information. Analysis scripts are available at https://github.com/martapn13/ProteomicsR (https://doi.org/10.5281/zenodo.19000356) and will be permanently archived upon publication.

## CRediT authorship contribution statement

**Marta Parente Nascimento:** Investigation, Writing – original draft, Methodology. **Ilyas Majid:** Formal analysis, Investigation, Writing – original draft, Validation. **Anu Arthy Murugan Karunagaran:** Investigation. **Sophia Ginns:** Investigation. **Dorothee Heger:** Investigation. **Cian Sutcliffe:** Investigation. **Ayesha Roked:** Methodology. **John McDonald:** Investigation. **Thong Huy Cao:** Methodology, Writing – review & editing. **Paulene Quinn:** Methodology. **Colleen B. Maxwell:** Methodology. **Luke A. Baker:** Investigation, Writing – review & editing. **Melanie J. Davies:** Funding acquisition. **Donald JL. Jones:** Methodology, Resources. **Leong L. Ng:** Validation, Methodology, Formal analysis. **Elina Akalestou:** Supervision, Funding acquisition, Investigation, Writing – original draft, Project administration.

## Funding

EA was supported by the BHF Leicester Centre of Research Excellence [RE/24/130031]. EA, MPN and AAMK were supported by a British Heart Foundation Accelerator Grant (AA/18/3/34220) and a Van Geest Heart and Cardiovascular Diseases Research Fund. IM and SG were supported by a BHF 4-year Multidisciplinary PhD Fellowship. T.H.C. was funded by the National Institute for Health and Care Research Leicester Biomedical Research Centre. The mass spectrometry work was supported by the John and Lucille Van Geest Foundation, and the National Institute for Health and Care Research Biomedical Research Centre Leicester.

## Declaration of competing interest

The authors declare that they have no known competing financial interests or personal relationships that could have appeared to influence the work reported in this paper.
