# Supplementary material for: Multi-organ proteomics identifies oxidative stress pathways underlying genetic and pharmacological SGLT2 suppression
Source: Redox Biol. 2026 Nov 3;98:104472. doi: 10.1016/j.redox.2026.104472 (PMC13760073; doi:10.1016/j.redox.2026.104472)
Supplement: Supplementary file 1 [file mmc1.docx]

Supp. Figure 1 **–** a. MCU, NDUFB10, TRIM63, DES and MUSTN1 gene expression in human primary isolated muscle cells treated with 0, 10nM and 10uM Dapagliflozin for 24hr, relative to UBC. b. Fold-change/ direction comparison of gene expression findings to proteomics analysis.

a

.

b

.

Supp. Figure 2 – KEGG biological pathways organised by human muscle and plasma, with the height of the bars and width of the lines connecting organs to KEGG annotations representing protein counts.

Supplementary Figure 3. Gene Ontology (GO) enrichment analysis of baseline differences in human skeletal muscle between the DAPA and DIET-CON groups. Differential protein abundance was assessed at baseline (week 0) using limma, and pathway enrichment analysis was subsequently performed on the resulting protein-level differences. Dot size indicates protein count and colour represents the adjusted P value.

Supp. Figure 4 – a – d. Gpx1 gene expression, e – h. Sod2 gene expression, i – l. Hmox1 gene expression, m – p. GSS gene expression and q – t. Prdx6 gene expression in liver, adrenals glands, White Adipose Tissue and Brown Adipose Tissue, in control, dapagliflozin-treated and SGLT2 null mice, relative to Ubc. * p <0.05

Supp. Figure 5 – 4HNE protein expression in liver tissue in control, dapagliflozin-treated and SGLT2 null mice.

Supp. Figure 6 – Total liver glutathione in liver tissue in control, dapagliflozin-treated and SGLT2 null mice. * p <0.05

Supp. Figure 7 – a. Aconitase N and b. aconitase C activity in Brown Adipose Tissue in control, dapagliflozin-treated and SGLT2 null mice. * p <0.05

Supp. Table 1 **-** Oxidative stress and redox-related Gene Ontology (GO) terms. A curated list of 114 GO oxidative stress and ROS related terms including Biological Process (BP), Molecular Function (MF), and Cellular Component (CC) ontologies to filter oxidative stress-related pathways.

| **GO ID** | **GO Term** | **Domain** |
| --- | --- | --- |
| GO:0006979 | response to oxidative stress | BP |
| GO:0034599 | cellular response to oxidative stress | BP |
| GO:0000302 | response to reactive oxygen species | BP |
| GO:0034614 | cellular response to reactive oxygen species | BP |
| GO:0070994 | response to reactive nitrogen species | BP |
| GO:1902882 | regulation of response to oxidative stress | BP |
| GO:1900409 | positive regulation of cellular response to oxidative stress | BP |
| GO:1900408 | negative regulation of cellular response to oxidative stress | BP |
| GO:1900407 | regulation of cellular response to oxidative stress | BP |
| GO:0016209 | antioxidant activity | MF |
| GO:0004096 | catalase activity | MF |
| GO:0004517 | nitric oxide synthase activity | MF |
| GO:0004602 | glutathione peroxidase activity | MF |
| GO:0006749 | glutathione metabolic process | BP |
| GO:0008631 | intrinsic apoptotic signaling pathway in response to oxidative stress | BP |
| GO:0042744 | hydrogen peroxide catabolic process | BP |
| GO:2000379 | regulation of reactive oxygen species metabolic process | BP |
| GO:1901031 | regulation of response to reactive oxygen species | BP |
| GO:0034613 | cellular response to reactive nitrogen species | BP |
| GO:0072593 | reactive oxygen species metabolic process | BP |
| GO:0034596 | cellular response to hydrogen peroxide | BP |
| GO:1902883 | negative regulation of response to oxidative stress | BP |
| GO:0006119 | oxidative phosphorylation | BP |
| GO:0022900 | electron transport chain | BP |
| GO:0006120 | mitochondrial electron transport NADH to ubiquinone | BP |
| GO:0006123 | mitochondrial electron transport cytochrome c to oxygen | BP |
| GO:0005743 | mitochondrial inner membrane | CC |
| GO:0005753 | mitochondrial proton transporting ATP synthase complex | CC |
| GO:0005739 | mitochondrion | CC |
| GO:0008137 | NADH dehydrogenase ubiquinone activity | MF |
| GO:0003954 | NADH dehydrogenase activity | MF |
| GO:0003950 | NAD plus ADP ribosyltransferase activity | MF |
| GO:0009060 | aerobic respiration | BP |
| GO:0033615 | mitochondrial respiratory chain complex IV assembly | BP |
| GO:0042775 | mitochondrial ATP synthesis coupled electron transport | BP |
| GO:0045259 | proton transporting ATP synthase activity | MF |
| GO:0043123 | positive regulation of I kappaB kinase NF kappaB signaling | BP |
| GO:0000165 | MAPK cascade | BP |
| GO:0035756 | cellular response to DNA damage stimulus | BP |
| GO:0016126 | sterol biosynthetic process | BP |
| GO:0070409 | cellular response to oxygen containing compound | BP |
| GO:0034440 | lipid oxidation | BP |

| GO:0034591 | cellular response to nitric oxide | BP |
| --- | --- | --- |
| GO:0046185 | aldehyde metabolic process | BP |
| GO:0016903 | oxidoreductase activity acting on aldehyde or oxo group | MF |
| GO:0004030 | aldehyde dehydrogenase NAD activity | MF |
| GO:0004029 | aldehyde dehydrogenase activity | MF |
| GO:0006577 | amino acid catabolic process | BP |
| GO:1990204 | cellular response to lipid hydroperoxide | BP |
| GO:0045454 | cell redox homeostasis | BP |
| GO:0098869 | cellular oxidant detoxification | BP |
| GO:0098754 | detoxification of reactive oxygen species | BP |
| GO:0019430 | removal of superoxide radicals | BP |
| GO:0004784 | superoxide dismutase activity | MF |
| GO:0004601 | peroxidase activity | MF |
| GO:0050660 | FAD binding | MF |
| GO:0006740 | NADPH metabolic process | BP |
| GO:0006741 | NADP metabolic process | BP |
| GO:0004364 | glutathione transferase activity | MF |
| GO:0004362 | glutathione disulfide reductase activity | MF |
| GO:0015035 | protein disulfide oxidoreductase activity | MF |
| GO:0015036 | disulfide oxidoreductase activity | MF |
| GO:0016667 | oxidoreductase activity acting on sulfur group donors | MF |
| GO:0045452 | regulation of cell redox homeostasis | BP |
| GO:0006281 | DNA repair | BP |
| GO:0006974 | cellular response to DNA damage stimulus | BP |
| GO:0045004 | DNA integrity checkpoint | BP |
| GO:0140694 | DNA damage response signal transduction | BP |
| GO:0006302 | double strand break repair | BP |
| GO:0006303 | double strand break repair via nonhomologous end joining | BP |
| GO:0034976 | response to endoplasmic reticulum stress | BP |
| GO:0035966 | protein oxidation | BP |
| GO:0006457 | protein folding | BP |
| GO:0034975 | protein folding in endoplasmic reticulum | BP |
| GO:0006986 | response to unfolded protein | BP |
| GO:0009063 | cellular amino acid catabolic process | BP |
| GO:0034592 | cellular response to lipid | BP |
| GO:0034593 | cellular response to fatty acid | BP |
| GO:0046486 | glycerolipid metabolic process | BP |
| GO:0097468 | lipid peroxidation involved in ferroptosis | BP |
| GO:1902175 | regulation of lipid peroxidation | BP |
| GO:0098868 | lipid hydroperoxide metabolic process | BP |
| GO:0034384 | lipid droplet organization | BP |
| GO:0035965 | ferroptosis | BP |
| GO:0044614 | response to oxidative damage | BP |
| GO:0006826 | iron ion transport | BP |
| GO:0005506 | iron ion binding | MF |
| GO:0034755 | iron ion homeostasis | BP |
| GO:0010042 | response to iron ion | BP |
| GO:0006879 | cellular iron ion homeostasis | BP |
| GO:0010282 | ferric iron binding | MF |
| GO:0015986 | ATP synthesis coupled proton transport | BP |
| GO:0032543 | mitochondrial translation | BP |
| GO:0098798 | mitochondrial protein complex assembly | BP |
| GO:0098799 | mitochondrial protein containing complex | CC |
| GO:0051881 | regulation of mitochondrial membrane potential | BP |
| GO:0000422 | autophagy of mitochondrion | BP |
| GO:0016175 | superoxide generating NADPH oxidase activity | MF |
| GO:0031408 | oxido reduction coenzyme metabolic process | BP |
| GO:0050664 | oxidoreductase activity acting on NAD(P)H | MF |
| GO:0052599 | reactive oxygen species biosynthetic process | BP |
| GO:0016651 | oxidoreductase activity acting on NADH or NADPH | MF |
| GO:0050661 | NADP binding | MF |
| GO:0071466 | cellular response to xenobiotic stimulus | BP |
| GO:0006805 | xenobiotic metabolic process | BP |
| GO:0006730 | one carbon metabolic process | BP |
| GO:0033554 | cellular response to stress | BP |
| GO:1990748 | cellular detoxification | BP |
| GO:0002755 | MyD88 dependent toll like receptor signaling pathway | BP |
| GO:0006919 | activation of cysteine type endopeptidase activity involved in apoptotic process | BP |
| GO:0071356 | cellular response to tumor necrosis factor | BP |
| GO:0071222 | cellular response to lipopolysaccharide | BP |
| GO:1901224 | regulation of innate immune response | BP |
| GO:0007204 | positive regulation of cytosolic calcium ion concentration | BP |

Supp. Table 2 – Primers used for the quantitative detection of transcripts normalized to UBC or Ubc.

| Target | Forward Sequence | Reverse Sequence |
| --- | --- | --- |
| *DES* | CCC TGA AGG GCA CTA ACG ATT | CTT CAG GGA GCA GTG AGG AC |
| *MCU* | CCA CCG GAC GGT ACA CCA | ACA TCA TTC AGC GTT GCT GC |
| *MUSTN1* | CTT CAG AGA CCC AGC GCT TA | GCC AGC TTG CTC ACA CTC T |
| *NDUFB10* | CGT TCG ACC TCA TCG TGG AC | CTT GGT CGA CTT TGT AGT CCC |
| *TRIM63* | GAG CCA CCT TCC TCT TGA CTG | GGG GCC TCT CAT TCA TCC AG |
| *UBC* | CAG CGG GATT TGG GTC G | CAC GAA GAT CTG CAT TGT CAA GT |
| *ACTINB* | TCC CTG GAG AAG AGC TAC GA | AGC ACT GTG TTG GCG TAC AG |
| *Gpx1* | CAG TTC GGA CAC CAG AAT GGC | GGT GGA AAG GCA TCG GGA AT |
| *Sod2* | GAA CAA TCT CAA CGC CAC CG | CTC CAG CAA CTC TCC TTT GGG |
| *Hmox1* | CAG AGC CGT CTC GAG CAT AG | CAA ATC CTG GGG CAT GCT GT |
| *Sod2* | GAA CAA TCT CAA CGC CAC CG | CTC CAG CAA CTC TCC TTT GGG |
| *Gss* | GAG CGA GTG GTG ACG TAT GC | CAT TGA GAA CGT GTC GGT GC |
| *Prdx6* | CGT ATC CGC TTC CAC GAT TTC C | TTC CT CTT GGC AAA CTC TGG CG |
| *Ubc* | CCA CAC AAA GCC CCT CAA TC | AAA GAT CTG CAT CGT CTC TCT C |
| *actinb* | CAC TGT CGA GTC GCG TCC | TCA TCC ATG GCG AAC TGG TG |

Supp. Table 3 – DETA trial patient characteristics

|  | *Mean ± SD* | |
| --- | --- | --- |
|  | *DAPA (n=11)* | *DIET (n=13)* |
| Sex (M/F) | 5/6 | 11/2 |
| Ethnicity (white / non-white) | 7/4 | 11/2 |
| Age (yr) | 65 ± 5 | 68 ± 7 |
| Height (cm) | 164 ± 12 | 172 ± 12 |
| Body mass (kg) | 88.1 ± 13.2 | 96.1 ± 18.3 |
| BMI (kg/m^2^) | 33.2 ± 5.5 | 32.5 ± 4.9 |
| Fat mass (kg) | 37.5 ± 9.5 | 36.5 ± 11.4 |
| Fat-free mass (kg) | 47.9 ± 9.8 | 56.3 ± 9.0* |
| VO_2peak_ (ml/kg/min) | 21.4 ± 4.4 | 23.0 ± 4.4 |
| Left handgrip (kg) | 25.0 ± 10.3 | 30.3 ± 7.7 |
| Right handgrip (kg) | 25.9 ± 10.5 | 33.1 ± 7.8 |
| HbA1c (%) | 7.8 ± 1.3 | 7.3 ± 1.3 |
| HbA1c (mmol) | 62.1 ± 13.8 | 55.8 ± 8.1 |
| eGFR (ml/min) | 76 ± 8 | 81 ± 8 |
| Duration of diabetes (yr) | 13.1 ± 8.0 | 12.5 ± 9.7 |
| Biguanide (Metformin) |  |  |
| n (%) | 9 (82%) | 9 (69%) |
| Mean duration, yr (SD) | 14.6 (8.2) | 14.8 (9.6) |
| Sulphonylurea |  |  |
| n (%) | 3 (27%) | 2 (15%) |
| Mean duration, yr (SD) | 14.4 (8.4) | 12.6 (6.9) |
| Statin |  |  |
| n (%) | 10 (91%) | 12 (92%) |
| Mean duration, yr (SD) | 12.9 (7.5) | 8.9 (8.5) |
| ACE inhibitor |  |  |
| n (%) | 8 (73%) | 5 (38%) |
| Mean duration, yr (SD) | 18.7 (9.8) | 9.6 (3.7) |
| Angiotensin receptor blocker (ARB) |  |  |
| n (%) | 2 (18%) | 3 (23%) |
| Mean duration, yr (SD) | 7.7 (1.4) | 16.7 (1.8) |
